# GPCR-A17 MAAP: mapping modulators, agonists, and antagonists to predict the next bioactive target

**DOI:** 10.1186/s13321-025-01050-z

**Published:** 2025-07-11

**Authors:** Ana B. Caniceiro, Ana M. B. Amorim, Nícia Rosário-Ferreira, Irina S. Moreira

**Affiliations:** 1 CNC-UC - Center for Neuroscience and Cell Biology, University of Coimbra, Rua Larga, Ed FMUC, Piso 1, 3004-504 Coimbra, Portugal; 2 CiBB - Centre for Innovative Biomedicine and Biotechnology, University of Coimbra, Rua Larga, Ed FMUC, Piso 1, 3004-504 Coimbra, Portugal; 3 Department of Life Sciences, University of Coimbra, Calçada Martim de Freitas, 3000-456 Coimbra, Portugal; 4 PURR.AI, IPN Incubadora, Rua Pedro Nunes, Ed C, 3030-199 Coimbra, Portugal; 5 Present Address: Lead contact, Coimbra, Portugal

**Keywords:** GPCR subfamily A17, Agonist, Antagonist, Modulator, Binding affinity (Ki), Ensemble learning, Drug discovery

## Abstract

G Protein-Coupled Receptors (GPCRs) are vital players in cellular signalling and key targets for drug discovery, especially within the GPCR-A17 subfamily, which is linked to various diseases. To address the growing need for effective treatments, the GPCR-A17 Modulator, Agonist, Antagonist Predictor (MAAP) was introduced as an advanced ensemble machine learning model that combines XGBoost, Random Forest, and LightGBM to predict the functional roles of agonists, antagonists, and modulators in GPCR-A17 interactions. The model was trained on a dataset of over 3,000 ligands (agonists, antagonists, and modulators) and 6,900 protein–ligand interactions, comprising all three ligand types, sourced from the Guide to Pharmacology, Therapeutic Target Database, and ChEMBL. It demonstrated a strong predictive performance, achieving F1 scores of 0.9179 and 0.7151, AUCs of 0.9766 and 0.8591, and specificities of 0.9703 and 0.8789, respectively, reflecting the overall performance across all classes in the testing and independent ligand validation datasets. A Ki-filtered subset of 4,274 interactions (where Ki is the inhibition constant that quantifies the ligand-binding affinity) improved the F1 scores to 0.9330 and 0.8267 for the testing and independent ligand datasets, respectively. By guiding experimental validation, GPCR-A17 MAAP accelerates drug discovery for various therapeutic targets. The code and data are available on GitHub (https://github.com/MoreiraLAB/GPCR-A17-MAAP).

**Graphical Abstract:**

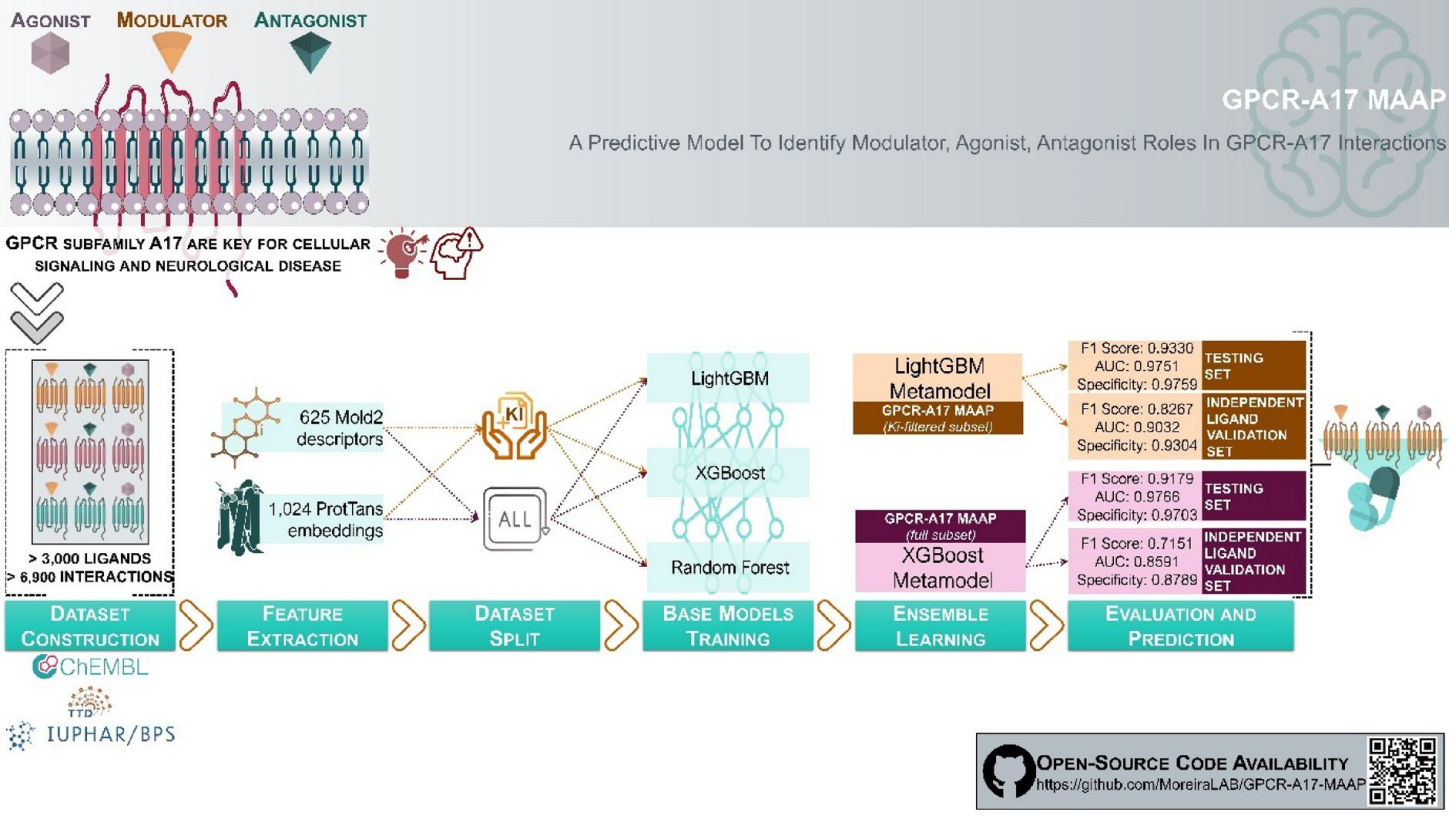

**Supplementary Information:**

The online version contains supplementary material available at 10.1186/s13321-025-01050-z.

## Scientific contribution

This research on GPCRs, particularly the GPCR-A17 subfamily, is significant because of their crucial role in cellular signalling and relevance in developing targeted therapies for complex health conditions. By advancing ligand-receptor interaction predictions (agonists, antagonists, and modulators), this study enhances our understanding of drug-receptor dynamics. These insights can streamline drug discovery, reduce experimental trial-and-error, and accelerate the identification of bioactive compounds for therapeutic applications.

**Supplementary Information:**

The online version contains supplementary material available at 10.1186/s13321-025-01050-z.

## Introduction

G Protein-Coupled Receptors (GPCRs) are the most abundant and versatile class of membrane proteins and are involved in numerous physiological processes by mediating extracellular signals in cellular responses [1, 2]. Among them, the GPCR-A17 subfamily includes a number of biogenic amine receptors, such as dopamine (D_1_R—D_5_R), serotonin (5-HT₂_A_, 5-HT₂_B_, 5-HT₂_C_, and 5-HT₆R), adrenergic α₁A, α₁B, α₁D, α₂A-C, β₁-β₃ receptors, and trace amine-associated receptors (TA_1_R, TA_2_R, TA_3_R, TA_5_R, TA_6_R, TA_8_R, and TA_9_R) [3, 4]. The activation of these receptors modulates cellular signalling, affecting cardiovascular function, metabolic regulation, immune response, and hormonal balance. Thus, dysregulation of the GPCR-A17 subfamily has broad implications for various physiological processes [4]. From a therapeutic standpoint, GPCR subfamily A17 receptors are interesting because agonists, antagonists, and allosteric modulators can be fine-tuned to regulate receptor signalling to restore normal physiology or mitigate pathological states. Agonists activate receptors by mimicking the actions of natural ligands. A well-known example is β_2_-adrenergic receptor agonists (e.g. albuterol), which help relax airway muscles, improve airflow, and alleviate breathing difficulties during asthma treatment [5–7]. Conversely, antagonists that block receptor activity without activating them are frequently employed to manage cardiovascular conditions, such as hypertension. For instance, β_1_-adrenergic receptor antagonists (e.g. atenolol) reduce heart rate and cardiac output by inhibiting excessive adrenergic signalling, thereby lowering blood pressure [6, 8, 9]. Modulators, also known as allosteric ligands, bind to non-orthosteric sites and do not fully activate or block receptors independently. Instead, they regulate receptor function by enhancing or diminishing the effects of agonists or antagonists [10].

Despite their therapeutic significance, ligand classification remains challenging because of the complexity of receptor-ligand interactions and the diverse functional responses they elicit [11–13]. A single receptor can interact with multiple ligands in distinct ways, leading to variable pharmacological effects, and a ligand that functions as an agonist for one receptor may act as an antagonist for another [14]. These variations are influenced by receptor-specific binding sites, conformational changes, and associated signalling pathways, underscoring the need for computational approaches that integrate both ligand and receptor features to improve the classification accuracy.

Machine Learning (ML) models have shown potential to assist in the classification of GPCR ligands, specifically between agonists and antagonists. Several models have been developed to predict this type of classification [15–21] by leveraging ligand properties, such as molecular structure and physicochemical characteristics, often represented as Simplified Molecular Input Line Entry System (SMILES) notations. Models such as Bayesian classifiers, Random Forest (RF), and Support Vector Machines (SVM) have demonstrated the ability to distinguish between agonists and antagonists [15, 18]. Recently, a two-step RF model achieved high accuracy by integrating molecular descriptors from pharmacological databases for virtual screening [19]. Although these approaches offer valuable insights, they primarily rely on ligand-based features and do not incorporate receptor sequence information, which is crucial for determining ligand behaviour across different GPCRs. DeepREAL, a deep learning framework, introduced a multiscale modelling approach that integrates protein sequence embeddings, chemical descriptors, and ligand-target interactions to enhance GPCR ligand classification [22]. However, it does not account for modulators, relies solely on neural network architectures, and lacks interpretability, which may limit its applicability in drug discovery.

Despite significant advancements in GPCR ligand classification, current models fail to account for modulators, an essential third class of ligands. To fill this gap, we developed a novel model designed to classify ligands as agonists, antagonists, and modulators, specifically for the GPCR subfamily A17 receptors, termed GPCR-A17 Modulators, Agonists, and Antagonist Predictors (MAAP). This model integrates the strengths of the three foundational models to enhance overall prediction performance. Notably, our approach incorporates previously overlooked key features, including receptor-specific data, which are crucial for improving the biological relevance of predictions. In a complementary analysis, we developed a Ki-filtered version of MAAP incorporating ligands with experimentally determined Inhibition Constant (Ki) values, which quantify binding affinity and provide insights into drug efficacy and safety [23]. However, its inclusion may introduce bias and potentially affect the model performance. We hypothesised that using Ki-filtered ligands, typically derived from controlled experiments [24, 25], would enhance the model accuracy by providing cleaner and more reliable data [26]. This filtering process reduces noise and inconsistencies, thereby improving robustness. Our dual-model strategy evaluates whether incorporating Ki enhances predictive accuracy or whether excluding it yields a more unbiased and stable model for GPCR-A17 ligand interactions.

We present two ensemble ML models, GPCR-A17 MAAP and GPCR-A17 MAAP (Ki-filtered), which leverage eXtreme Gradient Boosting (XGBoost) and Light Gradient-Boosting Machine (LightGBM) as metamodels, respectively. These ensembles were specifically designed to classify ligands as agonists, antagonists, or modulators of GPCR subfamily A17 receptors, combining the strengths of the base models to enhance the prediction performance. By incorporating both ligand- and receptor-based sequence features, the GPCR-A17 MAAP model addresses the need for a specialised tool where precise control over receptor signalling is critical for therapeutic success.

## Results

### Rationale behind choosing the best models

To select the three best methods for our models, we benchmarked a Deep Neural Network (DNN), XGBoost, LightGBM, K-Nearest Neighbour (KNN), Logistic Regression (LR), and RF, allowing each model to optimise its hyperparameters over 800 trials using Optuna [27]. The final model configurations, including the optimal hyperparameters, are presented in Table S1. The optimal hyperparameters for each method were selected by maximising the F1 score of the internal validation set, which was a randomly subsampled set used in the training phase. The corresponding F1 scores for each method are listed in Table S2. However, we could not rely solely on the F1 score from the internal validation set to identify the best-performing algorithms, because this set was used for hyperparameter tuning. Since the Optuna objective function was designed to maximise the F1 score during optimisation, there was a risk of overfitting the internal validation set. To mitigate this, we prioritised models that demonstrated better generalisation to the testing set, which contained unseen receptor-ligand pairs, although some ligands may have appeared in the training set. An independent ligand validation set was further used to evaluate and differentiate between the models. This additional evaluation ensured that the selected models not only performed well on the testing data, but also exhibited robustness when applied to unseen ligands, providing a more reliable measure of their generalisability.

### Models evaluation

After selecting the optimal hyperparameters for each method, we evaluated the performance of RF, DNN, XGBoost, LightGBM, KNN, and LR on what we refer to as the *full dataset* for consistency. This terminology was introduced to distinguish it from subsequent experiments conducted on a smaller Ki-filtered dataset, ensuring clarity in the analysis. Table 1 presents the performance of the RF, DNN, XGBoost, LightGBM, KNN, and LR *full datasets* across several key metrics, including the F1 score, precision, recall, Area Under the Curve (AUC), and specificity, on both the testing and independent ligand validation datasets. These metrics offer a comprehensive assessment of each model’s ability to effectively classify ligands as agonists, antagonists, or modulators, particularly considering the imbalanced nature of the dataset. This enabled us to determine the performance of each model across different ligand classes and its potential for future applications in GPCR-related drug discovery. Additionally, a detailed classification report for the independent ligand validation set, including precision, recall, F1 scores, and number of samples analysed for each class, was provided to enhance understanding (Table 2 for RF, XGBoost, and LightGBM, and Tables S3 to S5 for DNN, KNN, and LR *full datasets*, respectively).

**Table 1:** Performance of each method for the full dataset on the testing and independent ligand validation sets

Dataset	Method	Recall	Precision	F1 Score	AUC	Specificity
Testing	RF *full dataset*	0.9030 ± 0.0019	0.9042 ± 0.0019	0.9020 ± 0.0020	0.9689 ± 0.0002	0.9642 ± 0.0008
Independent ligand validation		0.7269 ± 0.0038	***0.7414 ± 0.0056***	0.6984 ± 0.0043	***0.8717 ± 0.0013***	0.8747 ± 0.0024
Testing	DNN *full* *dataset*	0.8648 ± 0.0073	0.8649 ± 0.0076	0.8637 ± 0.0075	0.9500 ± 0.0021	0.9479 ± 0.0032
Independent ligand validation		0.7064 ± 0.0067	0.6988 ± 0.0072	0.6934 ± 0.0086	0.8481 ± 0.0039	0.8614 ± 0.0045
Testing	XGBoost* full* *dataset*	0.9024 ± 0.0019	0.9034 ± 0.0019	0.9016 ± 0.0018	***0.9742 ± 0.0003***	0.9639 ± 0.0008
Independent ligand validation		*0.7335 ± 0.0043*	*0.7413 ± 0.0053*	0.7141 ± 0.0044	0.8697 ± 0.0008	***0.8789 ± 0.0027***
Testing	LightGBM *full dataset*	***0.9040 ± 0.0012***	***0.9051 ± 0.0012***	***0.9033 ± 0.0012***	0.9732 ± 0.0002	***0.9646 ± 0.0005***
Independent ligand validation		0.7324 ± 0.0053	0.7398 ± 0.0047	***0.7145 ± 0.0062***	0.8653 ± 0.0018	0.8782 ± 0.0033
Testing	KNN *full* *dataset*	0.8026 ± 0.0000	0.8024 ± 0.0000	0.8025 ± 0.0000	0.8912 ± 0.0000	0.9180 ± 0.0000
Independent ligand validation		0.7052 ± 0.0000	0.6997 ± 0.0000	0.6938 ± 0.0000	0.8270 ± 0.0000	0.8607 ± 0.0000
Testing	LR *full* *dataset*	0.8363 ± 0.0000	0.8361 ± 0.0000	0.8355 ± 0.0001	0.9246 ± 0.0000	0.9347 ± 0.0000
Independent ligand validation		0.6431 ± 0.0000	0.6267 ± 0.0000	0.6293 ± 0.0000	0.7694 ± 0.0000	0.8150 ± 0.0000

The best metrics are highlighted in bold for the testing set and in bold and italic for the independent ligand validation set to distinguish superior performance across both stages of evaluation. Data are presented as mean ± standard deviation. *RF* Random Forest, *DNN* Deep Neural Network, *XGBoost* Extreme Gradient Boosting, LightGBM Light Gradient Boosting Machine, *KNN* K-Nearest Neighbours, *LR* Logistic Regression

**Table 2:** RF, XGBoost, and LightGBM full dataset classification reports for each class in the independent ligand validation set

Dataset	Method	Recall	Precision	F1 Score	Number of Samples
Antagonist	RF *full dataset*	**0.9360 ± 0.0039**	0.6877 ± 0.0031	0.7928 ± 0.0020	350
	XGBoost *full dataset*	0.9040 ± 0.0053	0.7088 ± 0.0036	**0.7946 ± 0.0042**	
	LightGBM *full dataset*	0.8989 ± 0.0053	**0.7108 ± 0.0060**	0.7939 ± 0.0049	
Agonist	RF *full dataset*	0.7015 ± 0.0099	**0.8362 ± 0.0066**	**0.7629 ± 0.0084**	195
	XGBoost *full dataset*	**0.7231 ± 0.0073**	0.7851 ± 0.0074	0.7528 ± 0.0071	
	LightGBM *full dataset*	0.7159 ± 0.0070	0.7730 ± 0.0053	0.7433 ± 0.0061	
Modulator	RF *full dataset*	0.2626 ± 0.0111	0.7435 ± 0.0208	0.3878 ± 0.0104	147
	XGBoost *full dataset*	0.3415 ± 0.0117	0.7605 ± 0.0153	0.4713 ± 0.0134	
	LightGBM *full dataset*	**0.3578 ± 0.0159**	**0.7646 ± 0.0064**	**0.4873 ± 0.0144**	

The best metrics for each class are highlighted in bold font. Data are presented as mean ± standard deviation. *RF* Random Forest, *XGBoost* Extreme Gradient Boosting, *LightGBM* Light Gradient-Boosting Machine

### Performance analysis of an ensemble technique

During the model evaluation phase, the RF, XGBoost, and LightGBM *full datasets* were identified as the top three models because of their consistently high performances and complementary strengths. Subsequently, we applied an ensemble technique to assess its impact on performance across the testing and independent ligand validation sets. For each method, we predicted class probabilities in the internal validation set and combined them with the original feature space of the same dataset. Subsequently, three algorithms (RF, XGBoost, and LightGBM) were tested to determine the optimal metamodel. Table 3 lists the performance of each metamodel for the testing and independent ligand validation sets. Table 4 presents a detailed classification report for each metamodel in the independent ligand-validation set. From this perspective, we refer to blending ensemble methods as XGBoost, RF, or LightGBM metamodel *full datasets*.

**Table 3:** The performance of each algorithm (XGBoost metamodel full dataset, RF metamodel full dataset, and LightGBM metamodel full dataset) as a metamodel in the blending approach was evaluated using both the testing and independent ligand validation datasets

Dataset	Method	Recall	Precision	F1 Score	AUC	Specificity
Testing	XGBoost metamodel *full dataset*	**0.9181 ± 0.0010**	**0.9200 ± 0.0011**	**0.9179 ± 0.0010**	**0.9766 ± 0.0003**	**0.9703 ± 0.0004**
Independent ligand validation		*0.7335 ± 0.0023*	***0.7472 ± 0.0035***	0.7151 ± 0.0025	***0.8591 ± 0.0004***	0.8789 ± 0.0015
Testing	RF metamodel *full dataset*	0.9165 ± 0.0000	0.9180 ± 0.0000	0.9162 ± 0.0000	0.9760 ± 0.0000	0.9696 ± 0.0000
Independent ligand validation		**0.7370 ± 0.0000**	0.7443 ± 0.0000	***0.7222 ± 0.0000***	0.8463 ± 0.0000	***0.8810 ± 0.0000***
Testing	LightGBM metamodel *full dataset*	0.9159 ± 0.0008	0.9176 ± 0.0010	0.9156 ± 0.0010	0.9759 ± 0.0007	0.9694 ± 0.0003
Independent ligand validation		0.7283 ± 0.0047	0.7400 ± 0.0089	0.7102 ± 0.0047	0.8573 ± 0.0014	0.8756 ± 0.0030

The best metrics are highlighted in bold for the testing set and in bold font and italic for the independent ligand validation set to distinguish the superior performance across both stages of evaluation. Data are presented as mean ± standard deviation. *XGBoost*: Extreme Gradient Boosting, *RF*: Random Forest, *LightGBM*: Light Gradient Boosting Machine

**Table 4:** The classification report for each algorithm (XGBoost metamodel full dataset, RF metamodel full dataset, and LightGBM metamodel full dataset) as a metamodel in the blending approach was evaluated using an independent ligand validation dataset

Dataset	Method	Recall	Precision	F1 Score	Number of Samples
Antagonist	XGBoost metamodel *full dataset*	**0.9000 ± 0.0018**	0.7202 ± 0.0039	**0.8001 ± 0.0029**	350
	RF metamodel *full dataset*	0.8866 ± 0.0000	**0.7266 ± 0.0000**	0.7995 ± 0.0000	
	LightGBM metamodel *full dataset*	0.8931 ± 0.0078	0.7150 ± 0.0045	0.7942 ± 0.0043	
Agonist	XGBoost metamodel *full dataset*	0.7190 ± 0.0060	0.7317 ± 0.0021	0.7253 ± 0.0029	195
	RF metamodel *full dataset*	**0.7231 ± 0.0000**	**0.7382 ± 0.0000**	**0.7306 ± 0.0000**	
	LightGBM metamodel *full dataset*	0.7138 ± 0.0075	0.7319 ± 0.0030	0.7227 ± 0.0036	
Modulator	XGBoost metamodel *full dataset*	0.3565 ± 0.0054	**0.8319 ± 0.0178**	0.4991 ± 0.0083	147
	RF metamodel *full dataset*	**0.3946 ± 0.0000**	0.7945 ± 0.0000	**0.5273 ± 0.0000**	
	LightGBM metamodel *full dataset*	0.3551 ± 0.0100	0.8101 ± 0.0395	0.4934 ± 0.0122	

The best metrics are shown in bold font. Data are presented as mean ± standard deviation. *XGBoost* Extreme Gradient Boosting, *RF* Random Forest, *LightGBM* Light Gradient Boosting Machine

Based on the performance metrics shown in Table 3, we selected the XGBoost metamodel *full dataset* as the best ensemble model and named it GPCR-A17 MAAP. The model consistently demonstrated the best performance on the testing dataset, suggesting that the XGBoost metamodel was the most reliable predictor among the evaluated models for this dataset.

### Evaluation of the model filtered by Ki values

To rigorously assess the performance of the model when incorporating and filtering Ki values, we conducted a comprehensive evaluation to examine how the Ki values enhanced the predictive performance. By focusing on the Ki values, the model gained a sharper understanding of ligand-receptor interactions, resulting in more precise predictions of binding affinity. The best hyperparameters for the base models are listed in Table S1, and the best validation F1 scores (macro) during the hyperparameter tuning are presented in Table S6. We evaluated the Ki-filtered base model algorithms RF, DNN, KNN, LightGBM, XGBoost, and LR (Ki-filtered) on both the testing and independent ligand validation sets (Table S7); the corresponding classification reports are provided in Table S8.

We implemented an ensemble blending approach using XGBoost (Ki-filtered), LightGBM (Ki-filtered), and RF (Ki-filtered) base models. We then tested these algorithms as metamodels within the ensemble framework and further evaluated their performance on both the testing and independent ligand validation datasets. The results for the tested metamodels are listed in Table 5, and the corresponding classification results are presented in Table 6.

**Table 5:** The performance of each algorithm (XGBoost (Ki-filtered), RF (Ki-filtered), and LightGBM (Ki-filtered)) as a metamodel in the blending approach was evaluated using both the testing and independent ligand validation datasets

Dataset	Method	Recall	Precision	F1 Score	AUC	Specificity
Testing	XGBoost (Ki-filtered metamodel)	0.9304 ± 0.0010	0.9320 ± 0.0010	0.9308 ± 0.0010	**0.9761 ± 0.0002**	0.9751 ± 0.0004
Independent ligand validation		***0.8379 ± 0.0040***	***0.8365 ± 0.0041***	***0.8369 ± 0.0041***	***0.9084 ± 0.0003***	***0.9355 ± 0.0019***
Testing	RF (Ki- filtered metamodel)	0.9314 ± 0.0021	0.9314 ± 0.0021	0.9314 ± 0.0021	0.9751 ± 0.0005	0.9755 ± 0.0008
Independent ligand validation		0.8141 ± 0.0024	0.8123 ± 0.0024	0.8108 ± 0.0025	0.8893 ± 0.0016	0.9239 ± 0.0012
Testing	LightGBM (Ki-filtered metamodel)	**0.9325 ± 0.0000**	**0.9345 ± 0.0000**	**0.9330 ± 0.0000**	0.9751 ± 0.0004	**0.9759 ± 0.0000**
Independent ligand validation		0.8272 ± 0.0009	0.8264 ± 0.0006	0.8267 ± 0.0007	0.9032 ± 0.0004	0.9304 ± 0.0005

The best metrics are highlighted in bold for the testing set and in bold font and italic for the independent ligand validation set to distinguish the superior performance across both stages of evaluation. Data are presented as mean ± standard deviation. *XGBoost* Extreme Gradient Boosting, *RF* Random Forest, *LightGBM* Light Gradient Boosting Machine, *Ki* Inhibition Constant

**Table 6:** The classification report for each algorithm, XGBoost (Ki-filtered), RF (Ki-filtered), and LightGBM (Ki-filtered), as metamodels in the blending approach, was evaluated using an independent ligand validation dataset

Dataset	Method	Recall	Precision	F1 Score	Number of Samples
Antagonist	XGBoost (Ki-filtered metamodel)	0.8953 ± 0.0029	**0.8682 ± 0.0025**	**0.8815 ± 0.0020**	256
	RF (Ki-filtered metamodel)	**0.8984 ± 0.0025**	0.8262 ± 0.0024	0.8608 ± 0.0019	
	LightGBM (Ki-filtered metamodel)	0.8805 ± 0.0031	0.8669 ± 0.0023	0.8736 ± 0.0009	
Agonist	XGBoost (Ki-filtered metamodel)	**0.7608 ± 0.0120**	0.7901 ± 0.0093	**0.7752 ± 0.0106**	97
	RF (Ki-filtered metamodel)	0.6928 ± 0.0041	**0.8000 ± 0.0037**	0.7425 ± 0.0028	
	LightGBM (Ki-filtered metamodel)	0.7526 ± 0.0000	0.7511 ± 0.0091	0.7518 ± 0.0045	
Modulator	XGBoost (Ki-filtered metamodel)	**0.7405 ± 0.0054**	**0.7875 ± 0.0078**	**0.7632 ± 0.0036**	74
	RF (Ki-filtered metamodel)	0.6811 ± 0.0066	0.7802 ± 0.0057	0.7273 ± 0.0057	
	LightGBM (Ki-filtered metamodel)	0.7405 ± 0.0054	0.7851 ± 0.0012	0.7622 ± 0.0035	

The best metrics are in bold. Data are presented as mean ± standard deviation. *XGBoost* Extreme Gradient Boosting, *RF* Random Forest, *LightGBM* Light Gradient Boosting Machine, *Ki* Inhibition Constant

From this point onwards, we refer to LightGBM (Ki-filtered metamodel) as GPCR-A17 MAAP (Ki-filtered) because it was the best-performing Ki-filtered metamodel in the testing set. A summarised comparison of the XGBoost metamodel trained on the *full dataset* (GPCR-A17 MAAP) and Ki-filtered subset (GPCR-A17 MAAP (Ki-filtered)) is presented in Tables 7 and 8. In this comparison, we evaluated the impact of Ki-filtering to determine whether filtering the dataset improved model performance. The goal was to assess whether the Ki-filtered dataset, by creating a more consistent and reliable dataset and removing noisy or unreliable data, led to enhanced predictive performance compared with that using the *full dataset* without Ki-filtering. This analysis allowed us to understand whether the additional consistency gained from the Ki-filtered dataset provided tangible improvements in model performance.

**Table 7:** Performance comparison of the XGBoost metamodel full dataset (GPCR-A17 MAAP) with the LightGBM metamodel Ki-filtered subset (GPCR-A17 MAAP (Ki-filtered)) on the testing and independent ligand validation sets

Dataset	Method	Recall	Precision	F1 Score	AUC	Specificity
Testing	GPCR-A17 MAAP	0.9181 ± 0.0010	0.9200 ± 0.0011	0.9179 ± 0.0010	**0.9766 ± 0.0003**	0.9703 ± 0.0004
Independent ligand validation		0.7335 ± 0.0023	0.7472 ± 0.0035	0.7151 ± 0.0025	0.8591 ± 0.0004	0.8789 ± 0.0015
Testing	GPCR-A17 MAAP (Ki-filtered)	**0.9325 ± 0.0000**	**0.9345 ± 0.0000**	**0.9330 ± 0.0000**	0.9751 ± 0.0004	**0.9759 ± 0.0000**
Independent ligand validation		***0.8272 ± 0.0009***	***0.8264 ± 0.0006***	***0.8267 ± 0.0007***	***0.9032 ± 0.0004***	***0.9304 ± 0.0005***

The best metrics are highlighted in bold for the testing set and in bold and italic for the independent ligand validation set to distinguish superior performance across both stages of evaluation. Data are presented as mean ± standard deviation. *GPCR* G Protein-Coupled Receptor, *MAAP* modulator, agonist, and antagonist predictor, *Ki* Inhibition Constant

**Table 8:** Comparison between the classification reports of the full XGBoost metamodel dataset (GPCR-A17 MAAP) and the LightGBM metamodel Ki-filtered subset (GPCR-A17 MAAP (Ki-filtered)) for each class in the independent ligand validation set

Dataset	Method	Recall	Precision	F1 Score	Number of Samples
Antagonist	GPCR-A17 MAAP	**0.9000 ± 0.0018**	0.7202 ± 0.0039	0.8001 ± 0.0029	350
	GPCR-A17 MAAP (Ki-filtered)	0.8805 ± 0.0031	**0.8669 ± 0.0023**	**0.8736 ± 0.0009**	256
Agonist	GPCR-A17 MAAP	0.7190 ± 0.0060	0.7317 ± 0.0021	0.7253 ± 0.0029	195
	GPCR-A17 MAAP (Ki-filtered)	**0.7526 ± 0.0000**	**0.7511 ± 0.0091**	**0.7518 ± 0.0045**	97
Modulator	GPCR-A17 MAAP	0.3565 ± 0.0054	**0.8319 ± 0.0178**	0.4991 ± 0.0083	147
	GPCR-A17 MAAP (Ki-filtered)	**0.7405 ± 0.0054**	0.7851 ± 0.0012	**0.7622 ± 0.0035**	74

The best metrics for each class are highlighted in bold font. Data are presented as mean ± standard deviation. *GPCR* G Protein-Coupled Receptor, *MAAP* modulator, agonist and antagonist predictor, *Ki* Inhibition Constant

### Interpretation and feature contribution analysis

**Fig. 1:**
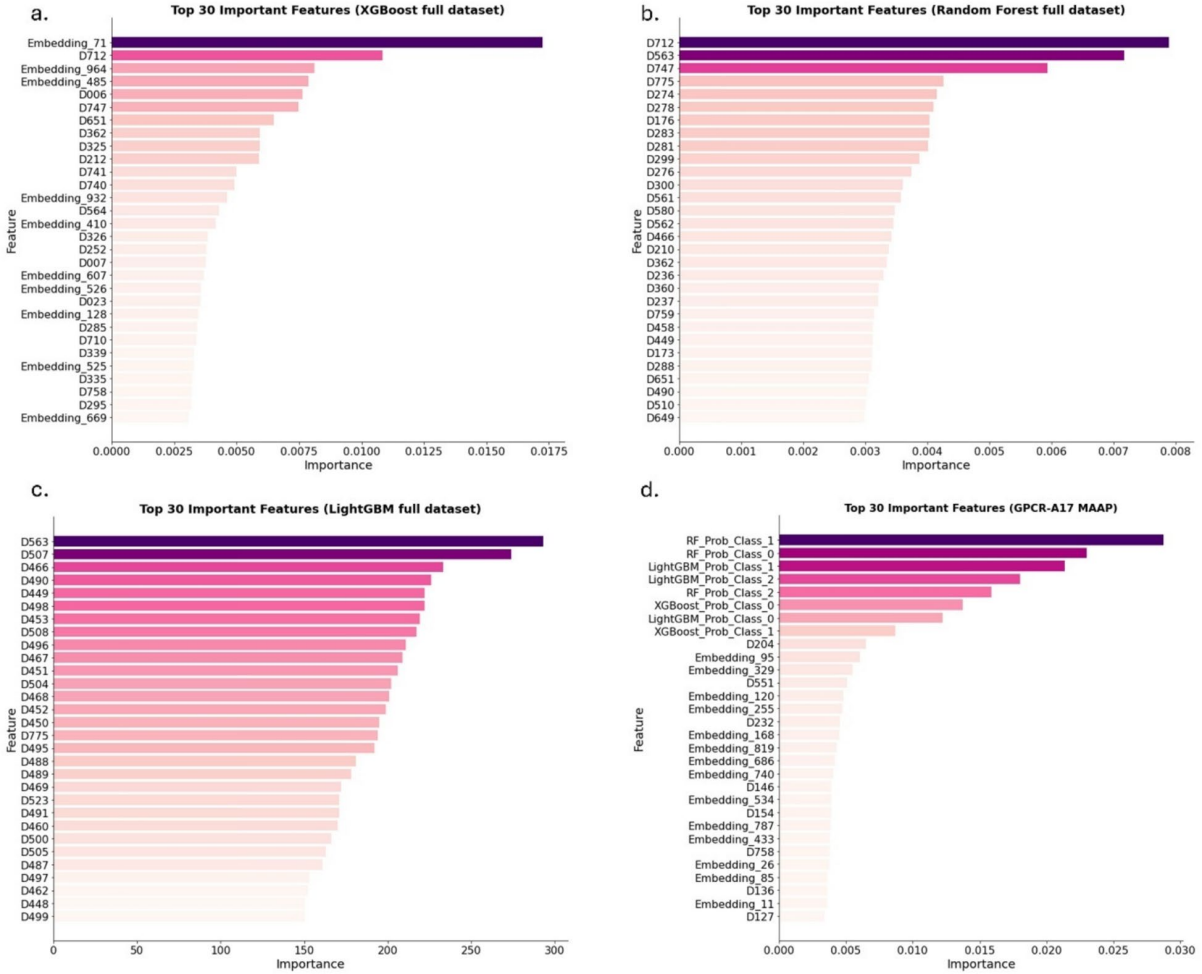
Feature importance plot for (**a**) XGBoost full dataset, (**b**) RF full dataset, (**c**) LightGBM full dataset base models, and (**d**) XGBoost metamodel full dataset (GPCR-A17 MAAP) illustrating the top 30 most important features. LightGBM: Light Gradient Boosting Machine. *RF* Random Forest, XGBoost: Extreme Gradient Boosting. The ligand features are listed in Table S9. [33]

**Fig. 2:**
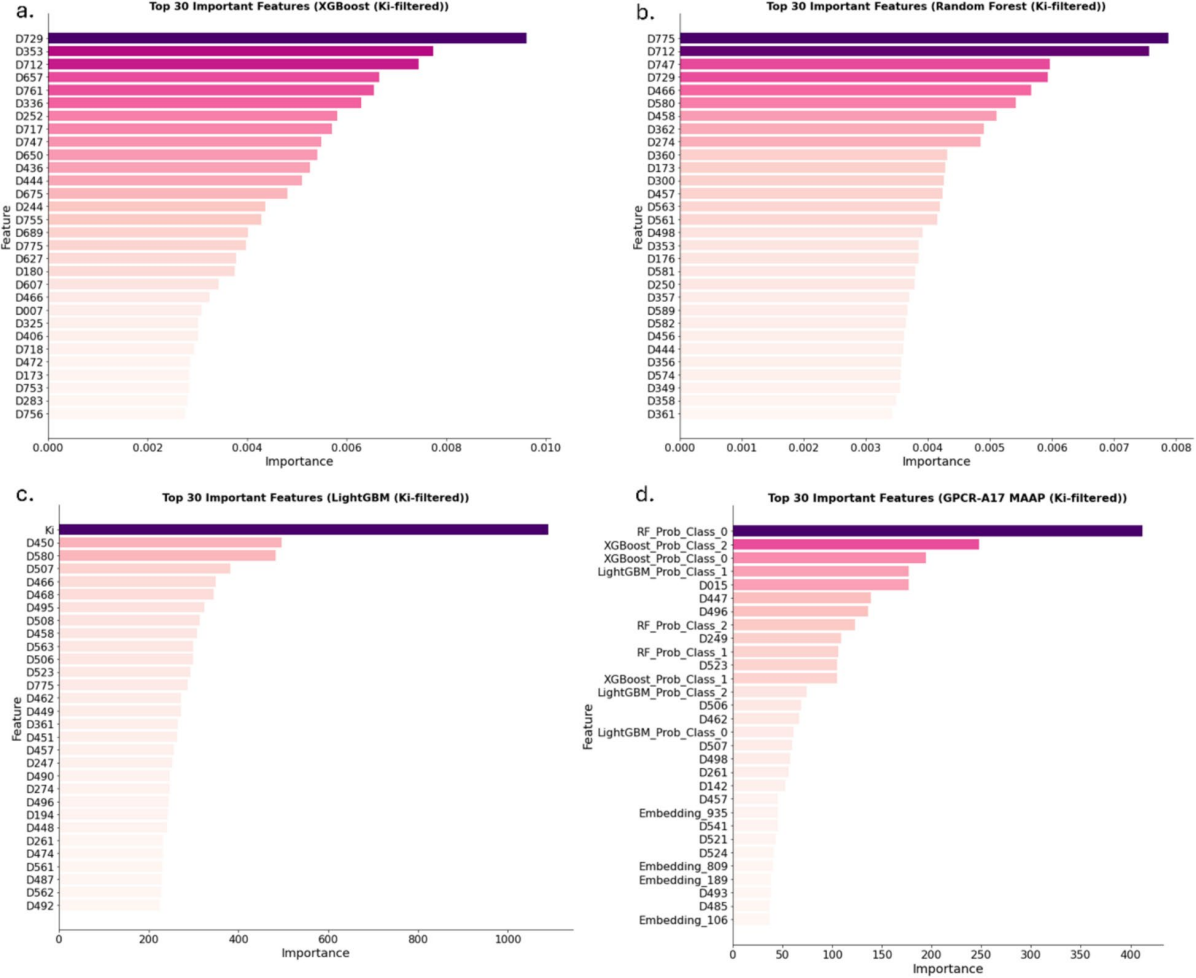
Feature importance plots for (**a**) XGBoost (Ki-filtered), (**b**) RF (Ki-filtered), (**c**) LightGBM (Ki-filtered) base models, and (**d**) LightGBM metamodel Ki-filtered (GPCR-A17 MAAP (Ki-filtered)) illustrate the top 30 most important features. *LightGBM* Light Gradient Boosting Machine. *RF* Random Forest, *XGBoost* Extreme Gradient Boosting, *Ki* Inhibition Constant. The ligand features are listed in Table S9 [33]

We present the results of the feature importance analysis for the *full dataset* base models and the best ensemble method, GPCR-A17 MAAP (XGBoost metamodel *full dataset*), as well as for the Ki-filtered base models and the best ensemble method, GPCR-A17 MAAP (Ki-filtered) (LightGBM metamodel Ki-filtered), to gain a deeper understanding of the contribution of each feature to the predictive performance of the model (Fig. 1 and 2). The predicted class probabilities from the base models are expected to play a crucial role in enhancing the model predictions within the ensemble learning framework. By examining these meta-features along with the original features such as protein embeddings and/or Ki values, we can better understand their relative influence on the output of the model.

## Discussion

GPCR-A17 MAAP is an XGBoost-based metamodel designed to predict whether a ligand functions as an antagonist, agonist, or modulator of various receptors within the GPCR-A17 subfamily. The macro F1 score was selected as the primary metric for optimising the objective function of the internal validation set and fine-tuning the hyperparameters within the Optuna framework. However, as previously mentioned, relying solely on the F1 score to select the best algorithm can lead to overfitting, because the model may become too specifically adapted to the internal validation set, hindering its ability to generalise to other datasets. The testing dataset, which was randomly sampled from the dataset but independent of the training process, was used to evaluate the generalisation capacity of each model. This shared presence of ligands between the training and testing sets arises from the fact that a single ligand can bind to multiple targets and a single target can interact with several ligands. Although the models were assessed using previously unseen target-ligand pairs, some ligands may have already been present in the training data. In contrast, the independent ligand validation dataset consisted exclusively of ligands not present during training, offering a more rigorous and realistic evaluation of the potential of the models to predict new compounds for drug-discovery applications. This approach provides a clearer measure of the robustness of the models and their ability to effectively generalise to truly novel data.

We tested six algorithms: LR, RF, LightGBM, XGBoost, KNN, and DNN *full dataset* on a dataset with 6,919 receptor-ligand pairs, achieving strong performance on the testing set and good performance on the independent ligand validation set. Among the six models evaluated, RF, XGBoost, and LightGBM emerged as top performers **(**Table 1**)**. The LightGBM *full dataset* model outperformed the XGBoost and RF *full datasets* in all metrics for the testing dataset (recall: 0.9040 ± 0.0012, precision: 0.9051 ± 0.0012, F1 score: 0.9033 ± 0.0012, specificity: 0.9646 ± 0.0005), except for AUC (0.9732 ± 0.0002). However, for the independent ligand validation dataset, the RF *full dataset* model achieved better precision (0.7414 ± 0.0056) and AUC (0.8717 ± 0.0013), whereas the LightGBM *full dataset* model achieved a better F1 score (0.7145 ± 0.0062), and the XGBoost *full dataset* achieved better recall (0.7335 ± 0.0043) and specificity (0.8789 ± 0.0027). Additionally, the DNN *full dataset* achieved one of the highest F1 scores on the internal validation set (Table S2), but underperformed on both the testing and independent ligand validation sets compared to other models, such as RF, XGBoost, and LightGBM (Tables 1 and S3). This discrepancy in the DNN performance can be attributed to the fact that DNNs typically excel when trained on large datasets, where they can fully capture complex patterns. In addition, the relatively small dataset used in this study made the DNN more prone to overfitting and less effective than other models tested. In contrast, traditional ML models such as XGBoost, LightGBM, and RF, which are more robust on smaller datasets, consistently outperform DNN. These models are less sensitive to the limitations of small datasets and less prone to overfitting, making them more suitable for this particular task. RF proved effective for high-dimensional data and demonstrated good generalisation owing to the structure of multiple decision trees trained on different data subsets. XGBoost, known for its efficient implementation of gradient boosting, stands out for its ability to minimise loss and enhance model performance through advanced regularisation techniques. LightGBM, recognised for its high speed and efficiency, also performed reliably on smaller datasets owing to its unique leafwise growth algorithm, which improved accuracy. Additionally, the LR and KNN *full dataset* models exhibited lower F1 scores: the LR *full dataset* achieved 0.8355 ± 0.0001 on the testing set and 0.6293 ± 0.0000 on the independent ligand validation set, whereas the KNN *full dataset* scored 0.8025 ± 0.0000 on the testing set and 0.6938 ± 0.0000 on the independent ligand validation set. The poor performance of these models can be attributed to their simplicity and inability to capture complex relationships in the data, rendering them unsuitable for this task.

Table 2 provides a detailed classification report of the three best-performing algorithms (RF, XGBoost, and LightGBM) for the independent ligand validation set, highlighting their ability to distinguish between different classes. The three models showed the strongest performance in classifying antagonists, likely because of the larger number of samples (350), which reflected a dataset imbalance. Following the antagonists, the models performed better in predicting the agonists and modulators. This performance aligns with sample availability, where agonists (195) were more represented than modulators (147), although the difference was less pronounced than that between antagonists and the other two classes. For the antagonist class, the RF *full dataset* achieved the highest recall (0.9360 ± 0.0039), the LightGBM *full dataset* achieved the highest precision (0.7108 ± 0.006), and the XGBoost full dataset achieved the highest F1 score (0.7946 ± 0.0042). For the agonist class, the RF *full dataset* achieved the highest precision (0.8362 ± 0.0066) and F1 score (0.7629 ± 0.0084), whereas the XGBoost *full dataset* recorded the highest recall (0.7231 ± 0.0073). Among the modulators, the LightGBM *full dataset* achieved the best recall (0.3578 ± 0.0159), precision (0.7646 ± 0.0064), and F1 score (0.4873 ± 0.0144). The drop in performance from the antagonist class to the modulator class reflected the imbalanced proportions of the dataset. These significant differences highlight the challenge posed by dataset imbalance, as models tend to perform better in overrepresented classes (e.g. antagonists) while struggling to predict underrepresented classes (e.g. modulators), resulting in biased performance. Although techniques, such as generating synthetic interactions between ligands and receptors, could potentially balance the dataset, these methods carry risks. Synthetic interactions may not accurately reflect real-world biological relationships, leading to misleading conclusions that the model performs well on synthetic data but fails to generalise to real drug interactions. Given these risks, we chose to proceed with the imbalanced dataset, ensuring that the models were trained on real and reliable interactions, despite the challenges posed by the imbalanced class performance.

Top-performing models (XGBoost, RF, and LightGBM *full datasets*) were selected for the ensemble methods. Despite the comparable performance across all metamodels (Table 3), the XGBoost *full dataset* metamodel consistently delivered the best metrics on the testing set and demonstrated better precision and AUC on the independent ligand validation set. Both the XGBoost and RF *full dataset* metamodels achieved strong performance across all classes **(**Table 4**).** Specifically, the XGBoost *full dataset* metamodel achieved the highest F1 score (0.8001 ± 0.0029) and recall (0.9000 ± 0.0018) for antagonists, as well as the highest precision (0.8319 ± 0.0178) for modulators. In contrast, the RF *full dataset* metamodel showed slightly better metrics for agonists, in addition to higher precision for antagonists (0.7266 ± 0.000), and recall (0.3946 ± 0.0000) and F1 score (0.5273 ± 0.0000) for modulators compared to the other models. Despite these nuances, the XGBoost *full dataset* metamodel demonstrated a more balanced and robust performance across both internal and external validations and was therefore selected as the final model, designated GPCR-A17 MAAP. Our approach began with a large and diverse dataset that provided a comprehensive chemical representation and trained the model to identify general patterns in ligand behaviour, specifically distinguishing between agonists, antagonists, and modulators. This initial broad dataset enabled the model to be generalised effectively across various ligand–protein interactions within the GPCR landscape.

To further enhance the precision of the model, we refined it using a smaller Ki-filtered dataset that included only ligands with reported Ki values. This refinement was not aimed at simply incorporating Ki as a feature but rather at leveraging data obtained under more consistent experimental conditions, where Ki values were reliably measured. Unlike other metrics, such as the half-maximal inhibitory concentration (IC50), Ki values are often derived from standardised experiments and provide a more dependable basis for training, thus reducing noise and improving model reliability [28]. Additionally, Ki values are biologically relevant indicators of binding affinity and play a crucial role in understanding the ligand-receptor interactions. By training the model on this refined subset, we ensured that it was exposed to data that accurately reflected true ligand-target interactions, minimising the risk of overfitting to any spurious patterns present in the broader dataset. This approach achieves a balance between broad generalisation from the initial dataset and precise, biologically relevant predictions from the refined dataset.

The procedure for the Ki-filtered dataset followed the same rigorous methodology used for the *full dataset* to ensure consistency in model evaluation and comparison. Similarly, the XGBoost (Ki-filtered), RF (Ki-filtered), and LightGBM (Ki-filtered) models exhibited the best performances (Table S7). Among these, LightGBM (Ki-filtered) achieved the highest metrics on the testing set (recall: 0.9278 ± 0.0010, precision: 0.9276 ± 0.0010, F1 score: 0.9277 ± 0.0010, specificity: 0.9741 ± 0.0004), whereas RF (Ki-filtered) achieved the best metrics on the independent ligand validation set (recall: 0.8215 ± 0.0027; precision: 0.8205 ± 0.0027; F1 score: 0.8171 ± 0.0026; AUC: 0.9022 ± 0.0011; specificity: 0.9276 ± 0.0014). RF (Ki-filtered) exhibited the highest performance metrics for the antagonist class (Table S8). For the agonist class, RF (Ki-filtered) had the highest precision (0.8403 ± 0.0046), whereas XGBoost (Ki-filtered) led to recall (0.6990 ± 0.0041) and F1 scores (0.7500 ± 0.0059). For the modulator class, RF (Ki-filtered) exhibited the highest precision (0.7395 ± 0.0157), XGBoost (Ki-filtered) exhibited the highest F1 score (0.7143 ± 0.0050), and LightGBM (Ki-filtered) exhibited the highest recall (0.7081 ± 0.0066). Following a similar approach, these three models were used to develop metamodels. The performance of the metamodels was similar to that of LightGBM (Ki-filtered metamodel) on the testing set and XGBoost (Ki-filtered metamodel) excelled on the independent ligand validation set **(**Table 5**)**. As expected, XGBoost (Ki-filtered metamodel) achieved the best metrics for classifying the modulator class, including the highest precision (0.8682 ± 0.0025), and outperformed the antagonist class with the best F1 score (0.8815 ± 0.0020). In addition, XGBoost (Ki-filtered metamodel) had the best recall (0.7608 ± 0.0120) and F1 score (0.7752 ± 0.0106) for the agonist class in the independent ligand validation dataset (Table 6). Based on our criterion that the model with the best metrics on the testing dataset was selected, the Ki-filtered metamodel (LightGBM) was chosen as the final model for this project involving Ki and named GPCR-A17 MAAP (Ki-filtered).

Both the GPCR-A17 MAAP and GPCR-A17 MAAP (Ki-filtered) models predicted whether a ligand acts as an antagonist, agonist, or modulator within the A17 subfamily of GPCR with high performance (Table 7). The key difference lies in the filtering of ligand–protein interactions with Ki values in the GPCR-A17 MAAP (Ki-filtered) model, along with the distinct metamodels used in each algorithm. However, the superior metrics observed in the Ki-filtered model cannot be solely attributed to the inclusion of Ki as a feature. Rather, it is the result of a more coherent dataset, where ligand-receptor interactions have been more rigorously studied and validated, as evidenced by the presence of Ki values in the literature. Consequently, the Ki-filtered model benefited from more consistent data, leading to better generalisation and improved classification accuracy (Table 8). In addition, the performance gap between the classes was more pronounced in the *full dataset*, further suggesting that the absence of filtering for Ki values may have introduced noise or unreliable data, particularly affecting these minority classes. By focusing on well-characterised interactions with Ki values, the model provides a more robust understanding of ligand-receptor binding dynamics, which enhances its predictive power beyond the simple classification of ligands as agonists, antagonists, or modulators.

As shown in Figs. 1 and 2**,** a feature analysis was conducted for the *full dataset* base models, GPCR-A17 MAAP, Ki-filtered base models, and GPCR-A17 MAAP (Ki-filtered). The model integrates protein embeddings with Mold2-derived ligand descriptors [29]. Feature importance analysis highlighted several descriptors that consistently influenced the model performance in datasets with and without experimentally determined Ki values. Among them, D712, which counts hydrogen bond donor atoms (N and O), was particularly prominent. As hydrogen bonding is critical for ligand-binding affinity and specificity [30], this descriptor likely reflects key molecular interactions. D747, which represents hydrogen bound to heteroatoms, is also related to this property and is frequently selected. Electronic features such as D563 and D551, corresponding to Burden matrix eigenvalues weighted by polarisabilities and Sanderson electronegativities, provide insight into the molecular electronic distribution, which is relevant for non-covalent interactions such as dipole or van der Waals forces. Topological descriptors, including D204 (vertex connectivity) and D232 (reciprocal distance index), capture branching and molecular compactness, which influence the fitting of ligands into binding pockets [31]. Autocorrelation features, such as D507 and D466, further describe the spatial organisation of atomic properties, shaping the ligand conformation and interaction profiles. In the Ki-filtered models, additional descriptors were important. D729 (number of = CHR groups) and D775 (hydrophilicity index) indicate the roles of unsaturation and polarity, whereas D015 (rotatable bond fraction) highlights the relevance of molecular flexibility for binding adaptability [32]. The recurrence of these descriptors across models supports their utility in capturing the meaningful structural and physicochemical patterns that govern ligand activity.

Both GPCR-A17 MAAP and GPCR-A17 MAAP (Ki-filtered) were performed as expected within the ensemble models, with the predicted probabilities from each base model dominating the feature importance ranking. Specifically, the probability of class 1 from RF emerged as the most significant feature in the GPCR-A17 MAAP, whereas the probability of class 0 from RF was the most important feature in the GPCR-A17 MAAP (Ki-filtered) model. Protein features (embeddings) play a key role in differentiating agonists, antagonists, and modulators, likely because they reflect receptor structures and binding sites. Since ligands can exhibit different actions across various receptors, protein features are crucial for accurately identifying the specific action of a ligand on a given receptor. These features provide an essential biological context, enabling models to better differentiate ligand-receptor interactions and improve predictions of ligand activity in specific receptor targets. Both GPCR-A17 MAAP and GPCR-A17 MAAP (Ki-filtered) models included protein features among the top 30 most important features (Fig. 1d and 2d). This aligns with the biological premise that ligand function is receptor-dependent and that receptor structural properties play a key role in defining activity. The Ki feature stands out primarily in the LightGBM (Ki-filtered) model, where it is ranked as the most important feature, suggesting a strong reliance on Ki values for prediction (Fig. 2c). However, this is an exception. In the other models, Ki did not feature prominently in the rankings; it appeared only in the 34th position in the RF (Ki-filtered) model (Fig. 2b) and did not rank within the top 30 features in XGBoost (Ki-filtered), where it was found at position 260th (Fig. 2a). This indicates that Ki did not significantly affect most models. In fact, in models such as GPCR-A17 MAAP (Ki-filtered), protein features are more prominent, reinforcing the idea that these models rely more on the underlying biological context of ligand-receptor interactions than on Ki values alone. Additionally, ligand features consistently dominate these rankings across models because of their direct relevance to ligand properties, which heavily influences interactions with GPCRs and thus impacts model predictions. These findings indicate that functional activity is influenced not only by affinity (Ki) but also by receptor-ligand interaction patterns and conformational dynamics. Additionally, Ki filtering improved the dataset quality, leading to better predictive performance, even in models in which Ki was not a major feature. This suggests that data curation, rather than the inclusion of Ki as a feature, was the main driver of performance improvements.

The GPCR-A17 MAAP ensemble model demonstrated a precision of 74,72% when presenting ligand-receptor interactions that were not included in the training set, indicating a high level of performance.

**Fig. 3:**
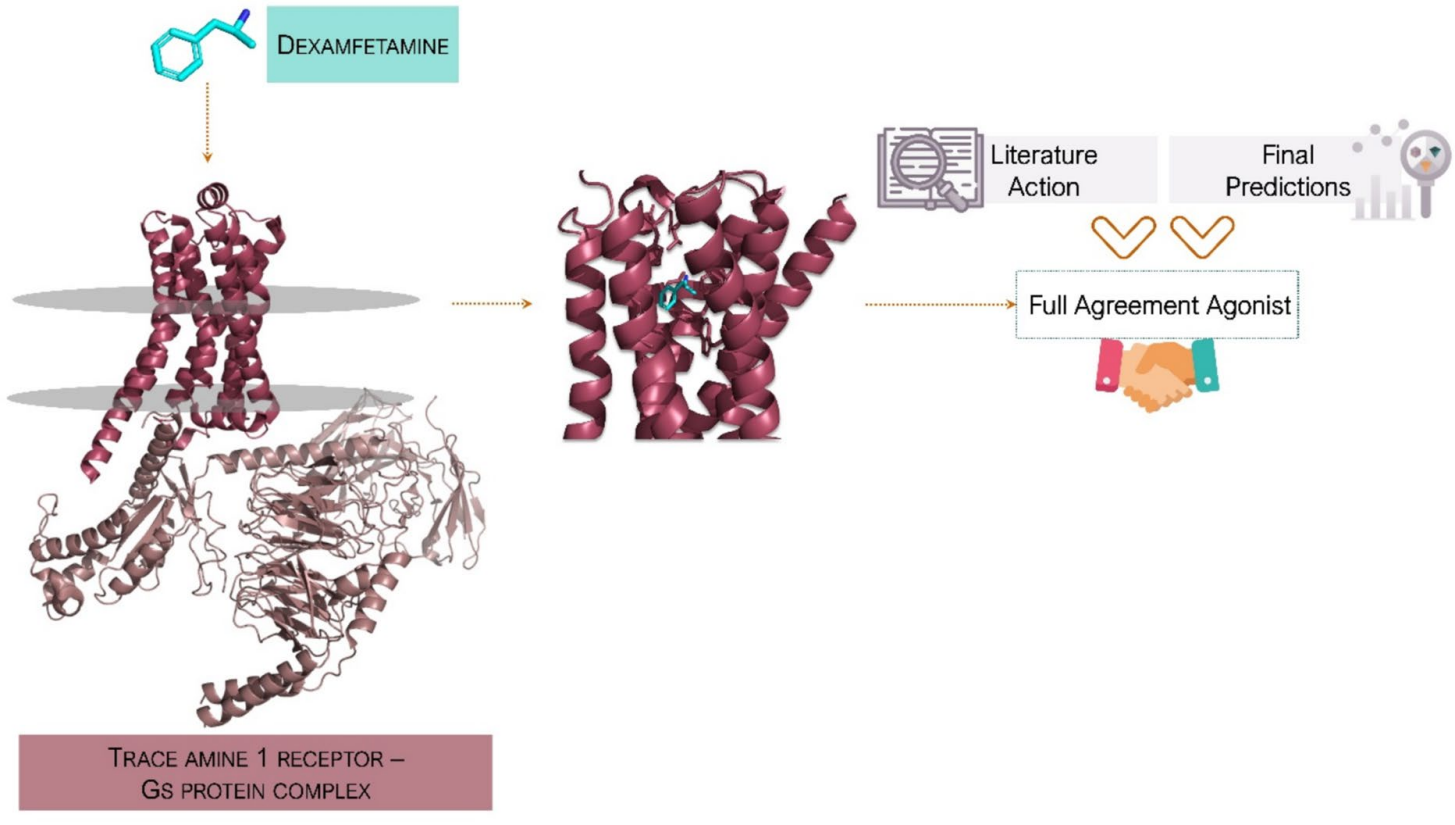
Structural interaction between Trace Amine-Associated Receptor 1 (TA1R) and dexamfetamine with agonistic activity prediction using the GPCR-A17 MAAP model. The figure shows the binding of dexamfetamine (cyan) within the binding pocket of TA1R (burgundy), coupled to a Gs protein complex (light pink) (PDB ID: 8JSO). The predicted action of the ligand as an agonist by the GPCR-A17 MAAP machine learning model (blue arrow) corresponds to the agonistic activity reported in the literature (burgundy arrow), thus validating the accuracy of the model

To illustrate this, we identified several correctly predicted interactions, such as the TA_1_R receptor complexed with the ligand dexamfetamine, whose activity was accurately predicted as an agonist (Fig. 3), consistent with previous reports [34, 35]. Similarly, the complex between α₁A adrenoceptor and the ligand clomipramine(a drug approved by the FDA) was correctly classified as an antagonist, in agreement with the known data [36]. Furthermore, our model correctly predicted the antagonistic activity of CHEMBL211301 binding to 5-HT_2A_ receptors, whereas all base models failed to make accurate predictions [37]. These examples highlight the strengths of the GPCR-A17 MAAP ensemble model and its potential as a reliable ligand classification tool.

Several ML models have been developed to classify ligands as either GPCR agonists or antagonists. However, benchmarking our model posed significant challenges because of issues related to dataset availability and the scope of the existing models. A primary limitation is that most datasets used in previous studies were restricted to classifying ligands as either agonists or antagonists. In contrast, our model adopts a more comprehensive approach by incorporating allosteric modulators in addition to agonists and antagonists. This expanded classification adds complexity, as the inclusion of modulators captures a broader range of ligand-receptor interactions than traditional models do not account for. Additionally, existing ML models are not designed to target the same GPCR subfamily as in our model. While our work focuses specifically on GPCR subfamily A17, the datasets used in most benchmarking studies pertain to different GPCR subfamilies. This mismatch in receptor subtypes creates a significant obstacle for benchmarking, as the ligands in these studies do not reflect the same receptor-ligand interactions relevant to our model. Consequently, a direct comparison between our model and existing models would be neither meaningful nor accurate because our model addresses a distinct and more complex biological landscape than existing models. To provide a meaningful benchmark within the scope of our study, we implemented two intuitive baseline methods: a per-receptor frequency-aware random classifier and a per-receptor similarity-based model. These baselines, described in detail in Supplementary Sect. 1, were evaluated using the same ligand validation sets used for model testing. As expected, both baselines underperformed compared to the GPCR-A17 MAAP and GPCR-A17 MAAP (Ki-filtered) models, particularly in their ability to generalise to novel ligand–receptor combinations. This comparative analysis reinforces the added value of using ML approaches that integrate diverse ligand and protein features to address the complexity of functional activity across GPCRs (Supplementary Sect. 1, Tables S10-S13).

The GPCR-A17 MAAP, which leverages protein features, achieved a strong performance, underscoring its potential as a highly effective model for predicting ligand-receptor interactions, particularly in future therapeutic applications. Additionally, GPCR-A17 MAAP (Ki-filtered), the best-performing method, further enhanced the predictive performance by incorporating Ki values, offering deeper insights into ligand-binding affinities and receptor interactions. The integration of both protein features with and without Ki values makes GPCR-A17 MAAP and GPCR- A17 MAAP (Ki-filtered) powerful tools for advancing GPCR-targeted drug discovery and therapeutic development.

## Conclusions

The GPCR-A17 MAAP model is a pioneering ensemble model designed to classify ligands as agonists, antagonists, or modulators of the GPCR subfamily A17 receptors. Importantly, many published models limit functional classification to binary tasks (e.g. agonist vs. antagonist) and often exclude modulators, a pharmacologically important class that is frequently overlooked in predictive modelling efforts [15, 17, 18, 20]. Our model addresses this gap by introducing a three-class functional classification system, enabling a more targeted and cost-effective approach to experimental validation than previous models. Unlike models designed to predict binding versus non-binding, an objective already addressed by established computational methods [38, 39], the GPCR-A17 MAAP focuses on determining the functional role of small molecules that are putative or predicted to be binders. This reflects a realistic scenario in early stage drug discovery, where candidate ligands are often identified through virtual screening, molecular docking, biophysical assays, or high-throughput screening, but their mechanisms of action remain unknown. By integrating receptor-specific data and employing a novel blending-based ensemble methodology, the model addresses key gaps in previous research and offers a specialised tool for studying ligand-receptor interactions.

A novel aspect of this model is its blending-based ensemble methodology, which significantly improves the accuracy and effectiveness of ligand classification compared with that of existing models. This approach has not been previously used in this domain. Another strength of this model is its reliance on sequence data rather than structural data, which are often limited owing to the scarcity of experimentally resolved protein–ligand complexes. By leveraging receptor sequences and SMILES ligands, the model can work with broader and more diverse datasets, making it more generalisable and robust. This capability is particularly valuable when structural data are incomplete or unavailable, thus expanding the applicability to a wider range of receptor-ligand interactions.

Although the Ki-filtered subset of the dataset yielded superior metrics, it is important to underscore the broader scope and utility of the full dataset model, which we designated the final GPCR-A17 MAAP model. The Ki-filtered model benefits from higher performance, partly owing to the more coherent and experimentally validated interactions in the subset. However, the full dataset model encompasses a larger and more diverse array of receptor-ligand interactions, including TA_3_R and TA_5_R, both members of the A17 subfamily. This diversity is critical for developing models that can generalise well across different receptors and ligands, beyond those for which Ki values are available. The ability to work with a broader range of interactions ensures that the GPCR-A17 MAAP model can be applied in various contexts, thereby extending its utility across GPCR subfamilies and therapeutic areas.

Despite its strengths, this model has some limitations. One limitation is dataset imbalance, with antagonists being overrepresented compared to agonists and modulators. This affects the model’s ability to generalise across all ligand categories, leading to a higher predictive performance for the majority class. To address this, we employed stratified k-fold cross-validation and imbalance-aware metrics (e.g. macro F1-score). However, it remains particularly challenging to classify modulators as accurately as the majority classes. Future improvements should prioritise increasing the representation of underrepresented ligand classes, such as modulators and agonists, as they become experimentally available, to enhance the classification performance across all ligand types. Another limitation is the model’s specificity to the GPCR-A17 receptor. While this enhances the biological relevance and predictive accuracy within this subfamily, it may limit direct generalisation to other GPCR subfamilies. Expanding this methodology to a broader range of GPCRs will be an important next step in increasing its applicability.

Despite these limitations, the GPCR-A17 MAAP methodology is adaptable and can be extended to other GPCR subfamilies and therapeutic targets with appropriate modifications. This flexibility enhances its relevance in drug discovery, particularly in identifying agonists, antagonists, and modulators, thereby offering valuable insights into ligand functions. Such insights are crucial because the same ligand may elicit different effects depending on the receptor subtype, influencing therapeutic strategies and clinical outcomes.

With its high classification precision, the GPCR-A17 MAAP represents a powerful tool for identifying novel therapeutic candidates, optimising current treatments, and supporting the development of targeted therapies. Furthermore, its ability to differentiate ligands across multiple categories deepens our understanding of ligand-receptor interactions, potentially accelerating the development of effective treatments for unmet medical needs.

### Experimental procedures

#### Data source and curation

To construct a comprehensive and representative dataset for training the models, we gathered data on ligands interacting with the GPCR-A17 subfamily from multiple sources, such as ChEMBL [40], Guide to Pharmacology [41], and Therapeutic Target Database (TTD) [42]. Focusing on bioactive molecules with drug-like properties, ChEMBL is a curated database that comprises genomic, bioactive, and chemical data [40]. The Guide to Pharmacology offers extensive information on biological drug targets and small molecules, including data on pharmacological targets, gene names, and ligand chemistry for the scientific and healthcare sectors [41]. The TTD provides extensive information on protein and nucleic acid therapeutic targets, their associated diseases, and the ligands that interact with them, including details of their mechanisms of action [42]. We specifically focused on ligands with reported actions on the GPCR-A17 receptors and categorised them into three functional groups: antagonists (represented as 0), agonists (represented as 1), and modulators (represented as 2). This classification is based on the effect of the ligand on receptor activity rather than structural differences. Antagonists are ligands that prevent receptor activation by agonists. This group includes both classical antagonists and inhibitors that block receptor function without triggering intrinsic signalling. Agonists include all ligands that directly modulate receptor signalling, either by increasing or decreasing activity. This category includes full, partial, inverse, and biased agonists. Although inverse agonists decrease receptor activity, they induce a functional response rather than simply blocking receptor activation, justifying their inclusion in this category. Modulators are ligands that bind outside the orthosteric site and alter receptor function through allosteric regulation. This category includes allosteric modulators, stimulators/enhancers, protein binders, and stabilisers. Given that some ligand annotations in databases can be ambiguous, we manually curated and refined the classifications to minimise mislabelling. Additionally, ligand-receptor interactions can be complex, as a single ligand may act on multiple GPCRs, and a single GPCR can bind to different ligands with varying effects. In cases where conflicting classifications were found for the same ligand-GPCR complex across different databases, we conducted a literature review to determine the most accurate classification, prioritising the primary signalling pathway of the receptor over others. This careful curation ensured that the dataset used for ML training and evaluation was accurate and consistent.

In total, the final dataset consisted of 6,919 GPCR-A17 receptor-ligand pairs, including 3,446 unique ligands and 21 unique GPCR-A17 receptors (D_1_R—D_5_R, 5-HT₂A, 5-HT₂B, 5-HT₂C, and 5-HT₆R, α₁A, α₁B, α₁D, α₂A-C, β₁-β₃, TA_1_R, TA_3_R, TA_5_R), with 3,677 classified as antagonists, 1,870 as agonists, and 1,372 as modulators.

### Ligand and protein feature extraction

To generate meaningful representations of both the ligands and GPCR-A17 receptor, relevant features that captured their bioactivity potential and structural characteristics were extracted. First, the SMILES were standardised using RDKit [43]. Ligand features were derived from SMILES using Mold2 [29], a tool that generates molecular descriptors that capture various chemical properties and encodes two-dimensional structural information of molecules. However, not all descriptors are useful for training the models. Descriptors with zero variance were excluded from the dataset because they did not contribute any discriminatory power, as they were constant across all data points and, therefore, could not distinguish between different ligands [44, 45]. After filtering, 625 descriptors remained (Table S9), each contributing unique information on the ligand properties. For GPCR-A17 receptors, we used ProtTrans [46], a cutting-edge DL model specifically designed to generate protein embeddings from the sequence data. These embeddings are high-dimensional vectors that capture the biophysical and functional properties of the protein sequences. A total of 1024 embeddings were calculated for each GPCR-A17 receptor. All ProtTrans embeddings were retained because they inherently encoded complex relationships within protein sequences. In addition, no embeddings exhibited zero variance or NaN values.

By combining the ligand descriptors and protein embeddings, we created a comprehensive feature vector for each ligand-receptor complex. The feature vector consisted of 1649 features, including 625 ligand-specific descriptors and 1,024 protein embedding descriptors. This feature vector effectively captures the bioactive properties of both the ligand and GPCR-A17 receptor as single entities. Using this enriched representation, the developed model can predict whether the ligand acts as an agonist, antagonist, or modulator based on these combined features.

### Data split and standardization

**Fig. 4:**
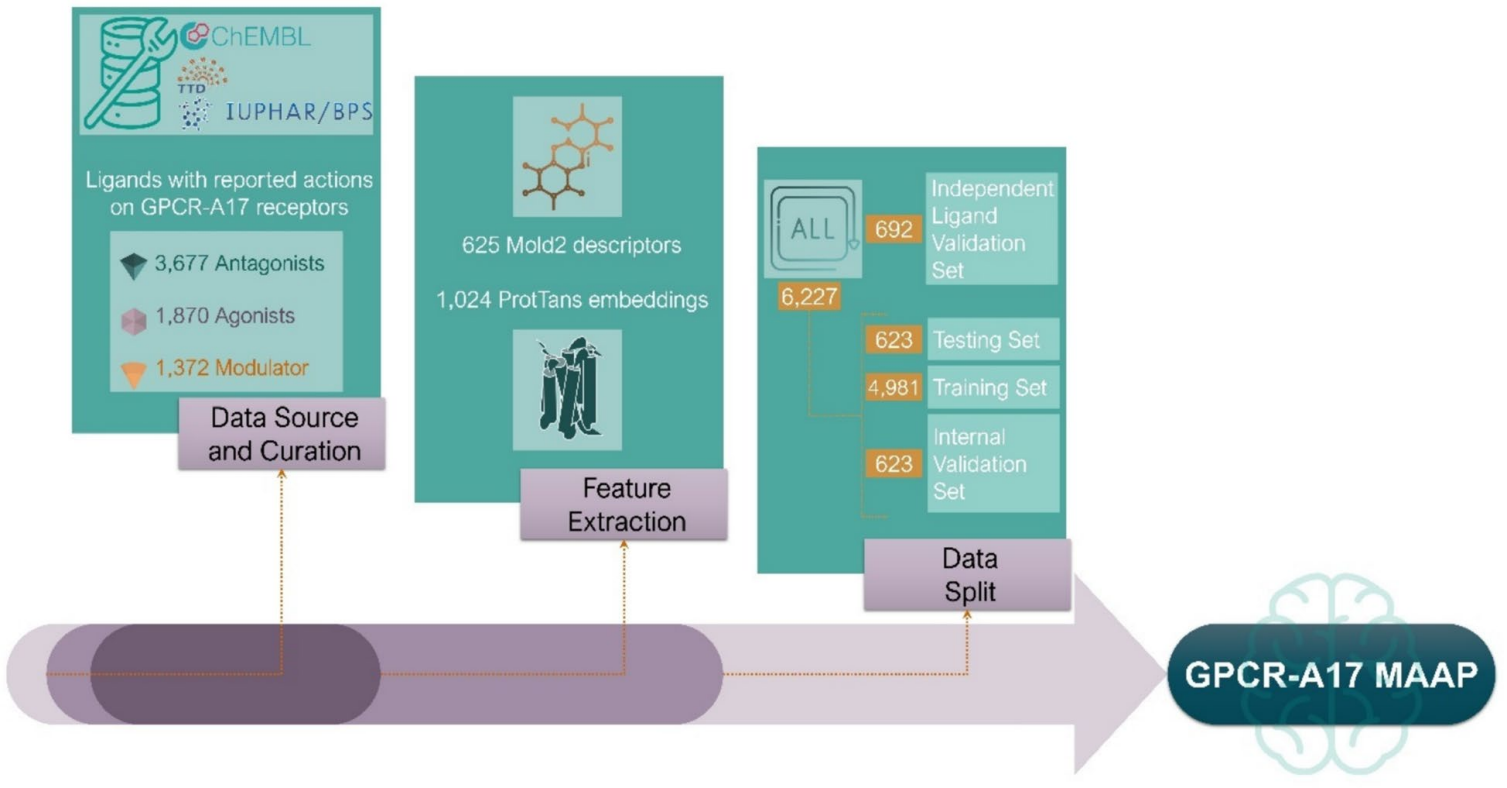
Overview of the dataset construction process. The key steps involved in constructing the dataset were data source, curation, feature extraction, and data splitting

To ensure a robust evaluation and performance of the models, we divided the dataset into distinct subsets for training, testing, and validation. First, 10% of the dataset (692 entries) was allocated to the independent ligand validation set, which consisted of ligands not included in the training, testing, or internal validation sets of the model. This separation is important for evaluating the ability of the model to generalise to entirely new ligands, providing a realistic measure of its performance on unseen data. The remaining 6,227 entries were split into training, testing, and internal validation sets, which were randomly divided as follows: 80% for training (4,981 entries), 10% for testing (623 entries), and 10% for internal validation (also 623 entries). This breakdown ensured that the model had sufficient data to learn the patterns (training set) while maintaining separate internal validation and testing sets to evaluate its performance during training and after tuning, respectively. The internal validation set aids in hyperparameter optimisation and prevents overfitting, whereas the testing set assesses how well the model generalises. However, both the testing and internal validation datasets may contain ligands previously observed during training, indicating that the model may have been partially exposed to these ligands. To address this, an independent ligand validation dataset was used to rigorously assess the generalisation of the model to novel compounds. Unlike the internal validation dataset used for hyperparameter tuning, this dataset is entirely distinct and essential in drug discovery. In the Results section, we present the model performance on the testing and independent validation sets (completely novel ligands). Figure 4 provides an overview of the dataset construction process, which includes data curation, feature extraction and data splitting.

To prepare the dataset for ML, we applied standardisation using the StandardScaler() function [47]. This method standardises the features by removing the mean and scaling the unit variance. Standardisation is important because it ensures that all features, regardless of their original scale, contribute equally to the model’s learning process, helping to improve model convergence and performance by preventing any single feature from dominating due to scale differences.

### Chemical diversity and scaffold analysis between datasets

To evaluate the model’s ability to generalise across diverse chemical spaces, we analysed scaffold diversity using the Bemis-Murcko scaffold approach [48], which retains core molecular structures while removing side chains. This assessment ensured that each dataset maintained adequate chemical diversity. We quantified scaffold diversity using total and unique scaffold counts, scaffold frequency distributions, and normalised Shannon entropy [49, 50], where higher values indicated greater structural diversity (Eqs. 1 and 2).

1 $${\varvec{H}}=\boldsymbol{ }-\boldsymbol{ }\sum_{{\varvec{i}}=1}^{{\varvec{M}}}{{\varvec{p}}}_{{\varvec{i}}}\boldsymbol{ }{{\varvec{l}}{\varvec{o}}{\varvec{g}}}_{2}({{\varvec{p}}}_{{\varvec{i}}})$$

2 $${{\varvec{H}}}_{{\varvec{n}}{\varvec{o}}{\varvec{r}}{\varvec{m}}{\varvec{a}}{\varvec{l}}{\varvec{i}}{\varvec{z}}{\varvec{e}}{\varvec{d}}}=\boldsymbol{ }\frac{{\varvec{H}}}{{{\varvec{l}}{\varvec{o}}{\varvec{g}}}_{2\boldsymbol{ }}{\varvec{M}}}$$

where H represents Shannon entropy, H_normalized_ is the normalised Shannon entropy (range 0–1), M is the number of unique scaffolds, p_i_ is the probability of occurrence of scaffold i, given by the count of scaffold i divided by the total count of all scaffolds, and log_2_M is the maximum entropy when all scaffolds are evenly distributed.

The independent ligand validation set was specifically curated to contain 63.92% novel scaffolds, ensuring exposure to previously unexplored chemical space. Although 36.08% of the scaffolds overlapped with the training set, the individual molecules (SMILES) in this set were distinct, enabling a rigorous model evaluation of the unseen compounds. To assess scaffold overlap between the datasets, we computed scaffold correlation scores (Figure S1). The training set shared a moderate scaffold overlap with the validation (0.097) and testing (0.13) sets, indicating that they originated from similar distributions. However, the independent ligand validation set exhibited a strong negative correlation (−0.59) with the training set, confirming its structural novelty and suitability for generalisation assessment. The scaffold distribution statistics for each dataset are summarised in Table S14, which includes the total scaffolds, unique scaffolds, scaffold frequency, and normalised Shannon entropy values. The training set had the highest number of scaffolds (4,981 total, 1297 unique), although its Unique/Total Ratio (0.2604) suggests scaffold redundancy. In contrast, the independent ligand validation set demonstrated the highest scaffold novelty (Unique/Total Ratio = 0.7009) and one of the most balanced scaffold distributions (Normalised Shannon Entropy = 0.9478). These characteristics make it the most challenging and rigorous testing set for assessing model generalisation beyond the training chemical space of the model.

### Models development

We selected six different algorithms to evaluate the model performance: DNN implemented using the TensorFlow package [51], XGBoost [52] implemented using the xgboost package, LightGBM [53] using the lightgbm package, and KNN [54], LR [55], and RF [56], both implemented using the scikit-learn package. Next, we conducted extensive hyperparameter tuning using the previously created training and internal validation datasets. We employed the Optuna [27] framework for an automated hyperparameter search, which is a powerful tool for determining optimal hyperparameters and benchmarking multiple algorithms for the same task. Optuna allows us to specify either the number of trials or the time limit for the optimisation process. It also enables the definition of an objective function to be optimised, which can involve either maximising or minimising a selected evaluation metric, such as the performance on an internal validation dataset [27]. A total of 800 trials were conducted for each method to identify the optimal hyperparameters using threefold stratified cross-validation [57] to ensure robust model evaluation and maximum performance. The decision to run the optimisation based on the number of trials rather than time was made to account for computational differences between the methods. This approach ensured consistent and sufficient trials for each model, leading to fair and comprehensive comparisons.

Given that the dataset was imbalanced, with more antagonists than agonists or modulators, we selected the F1 score (macro) as the primary metric to evaluate the model's ability to distinguish between the three classes. The F1 score balances both recall and precision, making it particularly effective for imbalanced datasets, where accuracy alone can be misleading. Specifically, we used the F1 score (macro), which calculates the F1 score independently for each class and then averages the results, ensuring that the performance across all classes is equally weighted regardless of the class size [58]. This approach was integrated into the objective function used by Optuna to maximise the F1 score (macro) of an independent internal validation dataset. This ensured that the model performed well across all ligand classes, particularly when managing class imbalances.

Table S1 provides the range of hyperparameters tested during the 800 trials of hyperparameter tuning for RF, XGBoost, LightGBM, DNN, KNN and LR.

### Model evaluation: statistical metrics

During hyperparameter tuning using the training and internal validation datasets, the F1 score (macro) was used to select the best model (Eq. 3). However, to ensure a more robust evaluation of the model performance on the testing and independent ligand validation datasets, we considered additional metrics beyond the F1 score (Eq. 4). Given the imbalanced nature of our dataset, we filtered the recall (Eq. 5), precision (Eq. 6), F1 score, specificity (Eq. 7), and AUC, which plots recall on the y-axis against the False Positive Rate (FPR) on the x-axis (Eq. 8). These metrics are more appropriate for evaluating the performance on imbalanced datasets, whereas metrics such as accuracy, which are more suitable for balanced datasets, were deprioritised. Recall, also known as sensitivity, measures a model’s ability to correctly identify all relevant positive instances. In contrast, precision evaluates the proportion of correct positive identifications. The AUC measures the model's ability to distinguish between classes, whereas specificity measures the model's ability to correctly identify negative instances [59]. In this context, (i) True Positives (TP) refer to instances in which the model correctly predicts positive cases; (ii) True Negatives (TN) represent cases in which the model correctly identifies negative cases; (iii) False Positives (FP) are cases in which the model incorrectly predicts positive cases when the actual class is negative; (iv) False Negatives (FN) are cases in which the model fails to identify a positive instance; and (v) FPR measures the proportion of false positives out of all actual negatives.

3 $${\varvec{F}}1 {\varvec{s}}{\varvec{c}}{\varvec{o}}{\varvec{r}}{\varvec{e}} ({\varvec{m}}{\varvec{a}}{\varvec{c}}{\varvec{r}}{\varvec{o}})= \frac{1}{{\varvec{N}}}{\sum }_{{\varvec{i}}=1}^{{\varvec{N}}}{\varvec{F}}1 {{\varvec{s}}{\varvec{c}}{\varvec{o}}{\varvec{r}}{\varvec{e}}}_{{\varvec{i}}}$$

4 $${\varvec{F}}1 {\varvec{s}}{\varvec{c}}{\varvec{o}}{\varvec{r}}{\varvec{e}} = 2 {\varvec{x}} \frac{{\varvec{P}}{\varvec{r}}{\varvec{e}}{\varvec{c}}{\varvec{i}}{\varvec{s}}{\varvec{i}}{\varvec{o}}{\varvec{n}} {\varvec{x}} {\varvec{R}}{\varvec{e}}{\varvec{c}}{\varvec{a}}{\varvec{l}}{\varvec{l}}}{{\varvec{P}}{\varvec{r}}{\varvec{e}}{\varvec{c}}{\varvec{i}}{\varvec{s}}{\varvec{i}}{\varvec{o}}{\varvec{n}} + {\varvec{R}}{\varvec{e}}{\varvec{c}}{\varvec{a}}{\varvec{l}}{\varvec{l}}}$$

5 $${\varvec{R}}{\varvec{e}}{\varvec{c}}{\varvec{a}}{\varvec{l}}{\varvec{l}} = \frac{{\varvec{T}}{\varvec{P}}}{{\varvec{T}}{\varvec{P}} + {\varvec{F}}{\varvec{N}}}$$

6 $${\varvec{P}}{\varvec{r}}{\varvec{e}}{\varvec{c}}{\varvec{i}}{\varvec{s}}{\varvec{i}}{\varvec{o}}{\varvec{n}} = \frac{{\varvec{T}}{\varvec{P}}}{{\varvec{T}}{\varvec{P}} + {\varvec{F}}{\varvec{P}}}$$

7 $${\varvec{S}}{\varvec{p}}{\varvec{e}}{\varvec{c}}{\varvec{i}}{\varvec{f}}{\varvec{i}}{\varvec{c}}{\varvec{i}}{\varvec{t}}{\varvec{y}} = \frac{{\varvec{T}}{\varvec{N}}}{{\varvec{T}}{\varvec{N}} + {\varvec{F}}{\varvec{P}}}$$

8 $${\varvec{F}}{\varvec{P}}{\varvec{R}} = \frac{{\varvec{F}}{\varvec{P}}}{{\varvec{F}}{\varvec{P}} + {\varvec{T}}{\varvec{N}}}$$

### Exploring ensemble technique

**Fig. 5:**
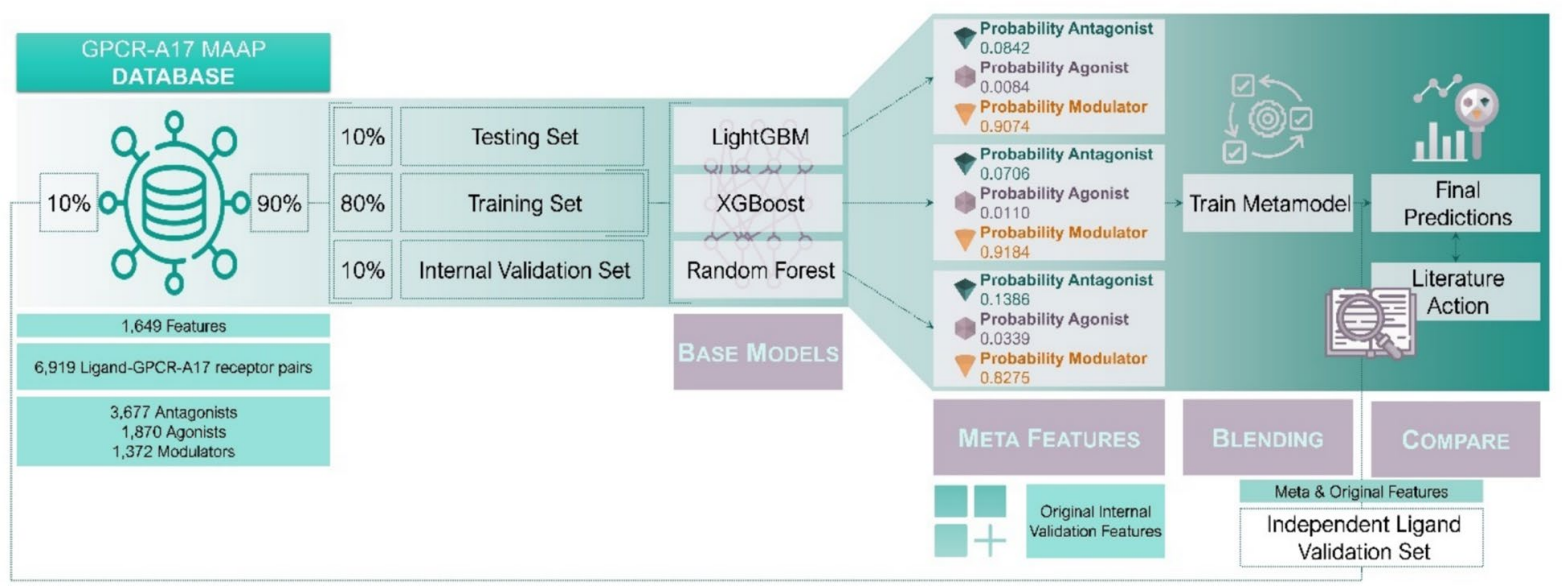
Overall blending process. Predictions from the top three models were used as meta-features, combined with the original features, and fed into a metamodel to generate a final prediction. This figure shows the process applied specifically to the independent ligand validation set to obtain final predictions

We investigated an ensemble technique, blending, using our top three best-performing models to evaluate and understand their combined effects on the overall model performance. The blending approach considers the predictions from the base models as meta-features and combines them with the original features to train a metamodel, which then produces the final prediction. In the blending approach, the top three best-performing models served as base models. We first predicted the class probabilities for each base model on the internal validation set using these probabilities as the meta-features for the metamodel. This produced nine columns corresponding to the predicted probabilities for each class from three different base model methods (one feature per class for each method). These meta-features were then combined with the original feature space of the internal validation set, which contained 1,649 features. Thus, the metamodel was trained on 1,658 features, leveraging both the original data and class probabilities from the base models. To make final predictions for the testing and independent ligand validation sets, we extracted meta-features from the predicted class probabilities of the base models and combined them with the original features of each dataset. We explored the top three best algorithms encountered previously as metamodels using the optimised hyperparameters identified through the Optuna optimisation process. Therefore, we selected the best algorithm to serve as the final metamodel. After generating the final predictions from the selected metamodel, we compared them with the actual outcomes and calculated the same set of performance metrics: F1 score, recall, precision, AUC, specificity, and classification reports for both the testing and independent ligand validation sets (Fig. 5). Additionally, we performed feature importance techniques to understand the influence of each feature in both the best base models (XGBoost, RF, and LightGBM) and the best ensemble model (XGBoost algorithm as a metamodel).

### Enhancing model performance with a Ki-filtered dataset

To optimise the predictive capabilities of our ML model for GPCR ligands, we first leveraged a large dataset and then refined our approach by incorporating a Ki-filtered subset. While we experimented with Ki as a feature, we ultimately prioritised using a Ki-filtered dataset rather than advocating Ki's inclusion as a feature for several key reasons: to reduce bias, improve data quality, and enhance model performance. The Ki-filtered subset, derived under controlled experimental conditions, provided a cleaner and more reliable dataset, leading to more robust prediction.

To construct a model with the Ki values as features, the dataset was carefully filtered and processed to ensure data integrity and reliability. This involved selection of ligands with reported Ki values and their corresponding actions. For ligand-target complexes with multiple Ki values, we applied a deduplication process to enhance data quality and consistency. This process involves calculating the z-score of each Ki value to identify and exclude outliers. Typically, Ki values with z-scores beyond a chosen threshold (e.g. |z|> 1) were considered outliers and were removed from the dataset. In cases where multiple Ki values for the same complex were very similar and had z-scores within the acceptable range (below the threshold), the Ki value with the z-score closest to zero (i.e. closest to the mean) was retained. This process ensured that only one representative Ki value, free of extreme deviations, was selected for each ligand-target complex. The z-score for each Ki value Kii was calculated as shown in Eq. (9), where (i) zi is the z-score of the Ki value Kii, (ii) Kii is the individual Ki value for a specific ligand-GPCR-A17 complex, (iii) μ is the mean of all Ki values for the corresponding ligand-GPCR-A17 complex, and (iv) σ is the standard deviation of the Ki values.

9 $${{\varvec{z}}}_{{\varvec{i}}} = \frac{{{\varvec{K}}{\varvec{i}}}_{{\varvec{i}}} - {\varvec{\mu}}}{{\varvec{\sigma}}}$$

The filtered dataset filtered 4,274 ligand-GPCR-A17 receptor entries, consisting of 2,009 unique ligands and 19 unique GPCR-A17 receptors (D_1_R—D_5_R, 5-HT₂_A_, 5-HT₂_B_, 5-HT₂_C_, 5-HT₆R, α₁A, α₁B, α₁D, α₂A-C, β₁-β₃ adrenoceptors, TA_1_R). Of these, 2,416 were classified as antagonists, 873 as agonists, and 985 as modulators. We used 10% of the data as the independent ligand validation set (427 entries) and split the remaining data into training (80%, 3,077 entries), testing (10%, 385 entries), and internal validation (10%, 385 entries) sets. Similar to the full dataset, we analysed the chemical diversity and scaffold distribution across datasets, as detailed in the section “Chemical Diversity and Scaffold Analysis Between Datasets”. The results were consistent with those of the full dataset, with 69.23% of the scaffolds in the Ki-filtered independent ligand validation set not being present in the training set. Additionally, the correlation between the training and independent ligand validation sets was −0.64, indicating a strong negative relationship between scaffold distributions (Figure S2). The high H_normalized_ values across all datasets further confirm chemical diversity, while the increased number of unique scaffolds across all Ki-filtered datasets, particularly in the independent ligand validation set, ensures that the model is evaluated on a broad and diverse scaffold space (Table S15). Furthermore, the independent ligand validation set served as a generalisation set for novel compounds, providing a realistic assessment of the predictive performance of the model for unseen SMILES.

We utilised Optuna once again to optimise the hyperparameters of the six tested algorithms, XGBoost, RF, LR, KNN, DNN, and LightGBM, using the same range of values for hyperparameter tuning as described in the "Model Development" section of Materials and Methods. We extracted ligand descriptors from Mold2 and protein descriptors from ProtTrans to ensure a comprehensive representation of both the chemical properties of the ligands and the structural characteristics of GPCRs. To enhance the quality of the dataset, we excluded descriptors with zero variance because they provided no discriminative value. Additionally, we incorporated the Ki value for each ligand-receptor complex, recognising its importance in quantifying ligand bioactivity. The integration of Ki values enhances the biological relevance of the dataset. Consequently, our final feature space comprised 1,637 features for each ligand-GPCR-A17 complex, providing a rich and detailed dataset that captures the key aspects of ligand-receptor interactions. The Ki value provides essential information regarding the strength of the interaction between the ligand and receptor, aiding in the differentiation between agonists, antagonists, and modulators. An internal validation set was used for hyperparameter optimisation to ensure the best performance of each model. After tuning, we evaluated the performance of the optimised models using both the testing and independent ligand validation sets. Following the same workflow as previously described, we implemented blending approaches, as illustrated in Fig. 5, using the newly optimised XGBoost, RF, and LightGBM base models. The metamodels were trained in a feature space that incorporated Ki values and consisted of 1,646 features. This feature space comprises the original 1,637 descriptors and class probabilities generated by the base models, providing a more comprehensive input for the metamodels. By integrating both the original features and predictive outputs of the base models, this approach aims to enhance the prediction performance and robustness, ensuring more accurate and reliable outcome ligands for receptor interactions.

### Explainability of models predictions

By analysing feature importance, we quantified how the Ki values and protein-specific features influenced the model predictions. This approach provides a more comprehensive understanding of the features that drive the model performance. In the Ki-filtered model, we examined the contribution of Ki values to performance compared with protein embeddings and other features, whereas in the model excluding Ki, we focused on the relative importance of protein features and other molecular characteristics. This comparative analysis between the models with and without Ki values allowed us to evaluate whether incorporating Ki added a significant predictive value or whether the model performed as well (or better) when relying solely on protein and other ligand features. The insights gained from this feature importance evaluation across the best individual models and their ensemble versions provided valuable guidance for optimising the prediction performance of the GPCR-A17 MAAP.

## Supplementary Information

Additional file 1.

## Data Availability

The datasets generated during this study are available at https://bit.ly/40uEb1x. All original code has been deposited at https://github.com/MoreiraLAB/GPCR-A17-MAAP.

## Abbreviations

AUC: Area under the curve
DNN: Deep neural network
FN: False negatives
FP: False positives
FPR: False positive rate
GPCR: G protein-coupled receptor
IC50: Half maximal inhibitory concentration
Ki: Inhibition constant
KNN: K-nearest neighbour
LightGBM: Light gradient-boosting machine
LR: Logistic regression
MAAP: Modulator, agonist and antagonist predictor
ML: Machine learning
RF: Random forest
SMILES: Simplified molecular input line entry system
SVM: Support vector machine
TN: True negatives
TP: True positives
TTD: Therapeutic target database
XGBoost: Extreme gradient boosting

## References

[1] Zhang M, Chen T, Lu X et al (2024) G protein-coupled receptors (GPCRs): advances in structures, mechanisms, and drug discovery. Signal Transduct Target Ther 9:88 38594257 10.1038/s41392-024-01803-6 PMC11004190

[2] Caniceiro AB, Bueschbell B, Schiedel AC, Moreira IS (2022) Class A and C GPCR dimers in neurodegenerative diseases. Curr Neuropharmacol 20:2081–2141 35339177 10.2174/1570159X20666220327221830 PMC9886835

[3] Joost P, Methner A (2002) Phylogenetic analysis of 277 human G-protein-coupled receptors as a tool for the prediction of orphan receptor ligands. Genome Biol 3:1 10.1186/gb-2002-3-11-research0063 PMC133447 12429062

[4] Caniceiro AB, Bueschbell B, Barreto CAV et al (2023) MUG: a mutation overview of GPCR subfamily A17 receptors. Comput Struct Biotechnol J 21:586–600 36659920 10.1016/j.csbj.2022.12.031 PMC9822836

[5] Baker JG (2010) The selectivity of beta-adrenoceptor agonists at human beta1-, beta2- and beta3-adrenoceptors. Br J Pharmacol 160:1048–1061 20590599 10.1111/j.1476-5381.2010.00754.x PMC2936015

[6] Baker JG (2005) The selectivity of beta-adrenoceptor antagonists at the human beta1, beta2 and beta3 adrenoceptors. Br J Pharmacol 144:317–322 15655528 10.1038/sj.bjp.0706048 PMC1576008

[7] De Pascali F, Ippolito M, Wolfe E et al (2022) β2 -Adrenoceptor agonist profiling reveals biased signalling phenotypes for the β2 -adrenoceptor with possible implications for the treatment of asthma. Br J Pharmacol 179:4692–4708 35732075 10.1111/bph.15900 PMC9474705

[8] Joseph SS, Lynham JA, Colledge WH, Kaumann AJ (2004) Binding of (-)-[3H]-CGP12177 at two sites in recombinant human beta 1-adrenoceptors and interaction with beta-blockers. Naunyn Schmiedebergs Arch Pharmacol 369:525–532 15060759 10.1007/s00210-004-0884-y

[9] Louis SN, Nero TL, Iakovidis D et al (1999) LK 204–545, a highly selective beta1-adrenoceptor antagonist at human beta-adrenoceptors. Eur J Pharmacol 367:431–435 10079020 10.1016/s0014-2999(99)00019-9

[10] Wang T (2013) The complexity of G-protein coupled receptor-ligand interactions. Sci China Chem 56:1344–1350

[11] Bueschbell B, Magalhães PR, Barreto CAV et al (2023) The World of GPCR dimers - Mapping dopamine receptor D2 homodimers in different activation states and configuration arrangements. Comput Struct Biotechnol J 21:4336–4353 37711187 10.1016/j.csbj.2023.08.032 PMC10497915

[12] Bueschbell B, Barreto CAV, Preto AJ et al (2019) A complete assessment of dopamine receptor- ligand interactions through computational methods. Molecules. 10.3390/molecules24071196 30934701 10.3390/molecules24071196 PMC6479630

[13] Kurose H, Kim SG (2022) Pharmacology of antagonism of GPCR. Biol Pharm Bull 45:669–674 35650094 10.1248/bpb.b22-00143

[14] Amorim AMB, Piochi LF, Gaspar AT et al (2024) Advancing drug safety in drug development: bridging computational predictions for enhanced toxicity prediction. Chem Res Toxicol 37:827–849 38758610 10.1021/acs.chemrestox.3c00352 PMC11187637

[15] Choi I-H, Kim H-J, Jung J-H et al (2010) Bayesian model for the classification of GPCR agonists and antagonists. Bull Korean Chem Soc 31:2163–2169

[16] Jabeen A, Ranganathan S (2019) Applications of machine learning in GPCR bioactive ligand discovery. Curr Opin Struct Biol 55:66–76 31005679 10.1016/j.sbi.2019.03.022

[17] Bushdid C, de March CA, Fiorucci S et al (2018) Agonists of G-protein-coupled odorant receptors are predicted from chemical features. J Phys Chem Lett 9:2235–2240 29648835 10.1021/acs.jpclett.8b00633 PMC7294703

[18] Ma C, Wang L, Xie X-Q (2011) Ligand classifier of adaptively boosting ensemble decision stumps (LiCABEDS) and its application on modeling ligand functionality for 5HT-subtype GPCR families. J Chem Inf Model 51:521–531 21381738 10.1021/ci100399j PMC3065508

[19] Oh J, Ceong H-T, Na D, Park C (2022) A machine learning model for classifying G-protein-coupled receptors as agonists or antagonists. BMC Bioinform 23:346 10.1186/s12859-022-04877-7 PMC9389651 35982407

[20] Zhu X-L, Cai H-Y, Xu Z-J et al (2011) Classification of 5-HT(1A) receptor agonists and antagonists using GA-SVM method. Acta Pharmacol Sin 32:1424–1430 21963891 10.1038/aps.2011.112 PMC4002729

[21] Caniceiro AB, Orzeł U, Rosário-Ferreira N et al (2025) Leveraging artificial intelligence in GPCR activation studies: computational prediction methods as key drivers of knowledge. Methods Mol Biol 2870:183–220 39543036 10.1007/978-1-0716-4213-9_10

[22] Cai T, Abbu KA, Liu Y, Xie L (2022) DeepREAL: a deep learning powered multi-scale modeling framework for predicting out-of-distribution ligand-induced GPCR activity. Bioinformatics 38:2561–2570 35274689 10.1093/bioinformatics/btac154 PMC9048666

[23] Copeland RA, Pompliano DL, Meek TD (2006) Drug-target residence time and its implications for lead optimization. Nat Rev Drug Discov 5:730–739 16888652 10.1038/nrd2082

[24] Du X, Li Y, Xia Y-L et al (2016) Insights into protein-ligand interactions: Mechanisms, models, and methods. Int J Mol Sci 17:144 26821017 10.3390/ijms17020144 PMC4783878

[25] Bernetti M, Cavalli A, Mollica L (2017) Protein-ligand (un)binding kinetics as a new paradigm for drug discovery at the crossroad between experiments and modelling. Medchemcomm 8:534–550 30108770 10.1039/c6md00581k PMC6072069

[26] Gaulton A, Bellis LJ, Bento AP et al (2012) ChEMBL: a large-scale bioactivity database for drug discovery. Nucleic Acids Res 40:D1100–D1107 21948594 10.1093/nar/gkr777 PMC3245175

[27] Akiba T, Sano S, Yanase T, et al (2019) Optuna. In: Proceedings of the 25th ACM SIGKDD International Conference on Knowledge Discovery & Data Mining. ACM, New York, NY, USA

[28] Burlingham BT, Widlanski TS (2003) An intuitive look at the relationship of ki and IC50: a more general use for the Dixon plot. J Chem Educ 80:214

[29] Hong H, Xie Q, Ge W et al (2008) Mold(2), molecular descriptors from 2D structures for chemoinformatics and toxicoinformatics. J Chem Inf Model 48:1337–1344 18564836 10.1021/ci800038f

[30] Chen D, Oezguen N, Urvil P et al (2016) Regulation of protein-ligand binding affinity by hydrogen bond pairing. Sci Adv 2:e1501240 27051863 10.1126/sciadv.1501240 PMC4820369

[31] Amić D, Bešlo D, Lučić B et al (1998) The vertex-connectivity index revisited. J Chem Inf Comput Sci 38:819–822

[32] Qi S, Krogsgaard M, Davis MM, Chakraborty AK (2006) Molecular flexibility can influence the stimulatory ability of receptor-ligand interactions at cell-cell junctions. Proc Natl Acad Sci U S A 103:4416–4421 16537380 10.1073/pnas.0510991103 PMC1450186

[33] (2024) Access Mold2. In: U.S. Food & Drug. https://www.fda.gov/science-research/mold2/access-mold2. Accessed 4 Oct 2024

[34] Barak LS, Salahpour A, Zhang X et al (2008) Pharmacological characterization of membrane-expressed human trace amine-associated receptor 1 (TAAR1) by a bioluminescence resonance energy transfer cAMP biosensor. Mol Pharmacol 74:585–594 18524885 10.1124/mol.108.048884 PMC3766527

[35] Lewin AH, Miller GM, Gilmour B (2011) Trace amine-associated receptor 1 is a stereoselective binding site for compounds in the amphetamine class. Bioorg Med Chem 19:7044–7048 22037049 10.1016/j.bmc.2011.10.007 PMC3236098

[36] Proudman RGW, Pupo AS, Baker JG (2020) The affinity and selectivity of α-adrenoceptor antagonists, antidepressants, and antipsychotics for the human α1A, α1B, and α1D-adrenoceptors. Pharmacol Res Perspect 8:e00602 32608144 10.1002/prp2.602 PMC7327383

[37] McLean TH, Chambers JJ, Parrish JC et al (2006) C-(4,5,6-trimethoxyindan-1-yl)methanamine: a mescaline analogue designed using a homology model of the 5-HT2A receptor. J Med Chem 49:4269–4274 16821786 10.1021/jm060272y

[38] Latek D, Prajapati K, Dragan P et al (2025) GPCRVS - AI-driven decision support system for GPCR virtual screening. Int J Mol Sci. 10.3390/ijms26052160 40076783 10.3390/ijms26052160 PMC11900134

[39] Chan WKB, Zhang Y (2020) Virtual screening of human Class-A GPCRs using ligand profiles built on multiple ligand-receptor interactions. J Mol Biol 432:4872–4890 32652079 10.1016/j.jmb.2020.07.003 PMC7415681

[40] Zdrazil B, Felix E, Hunter F et al (2024) The ChEMBL Database in 2023: a drug discovery platform spanning multiple bioactivity data types and time periods. Nucleic Acids Res 52:D1180–D1192 37933841 10.1093/nar/gkad1004 PMC10767899

[41] Harding SD, Sharman JL, Faccenda E et al (2018) The IUPHAR/BPS Guide to PHARMACOLOGY in 2018: updates and expansion to encompass the new guide to IMMUNOPHARMACOLOGY. Nucleic Acids Res 46:D1091–D1106 29149325 10.1093/nar/gkx1121 PMC5753190

[42] Zhou Y, Zhang Y, Lian X et al (2022) Therapeutic target database update 2022: facilitating drug discovery with enriched comparative data of targeted agents. Nucleic Acids Res 50:D1398–D1407 34718717 10.1093/nar/gkab953 PMC8728281

[43] Landrum G, Tosco P, Kelley B, et al (2024) rdkit/rdkit: 2024_03_6 (Q1 2024) Release. Zenodo

[44] Preto AJ, Caniceiro AB, Duarte F et al (2024) POSEIDON: Peptidic Objects SEquence-based Interaction with cellular DOmaiNs: a new database and predictor. J Cheminform 16:18 38365724 10.1186/s13321-024-00810-7 PMC10874016

[45] Sarker IH (2021) Machine learning: Algorithms, real-world applications and research directions. SN Comput Sci 2:160 33778771 10.1007/s42979-021-00592-x PMC7983091

[46] Elnaggar A, Heinzinger M, Dallago C et al (2022) ProtTrans: Toward understanding the language of life through self-supervised learning. IEEE Trans Pattern Anal Mach Intell 44:7112–7127 34232869 10.1109/TPAMI.2021.3095381

[47] StandardScaler. In: scikit-learn. https://scikit-learn.org/stable/modules/generated/sklearn.preprocessing.StandardScaler.html. Accessed 20 Sep 2024

[48] Bemis GW, Murcko MA (1996) The properties of known drugs. 1 Molecular frameworks. J Med Chem 39:2887–2893 8709122 10.1021/jm9602928

[49] Guha R, Velegol D (2023) Harnessing Shannon entropy-based descriptors in machine learning models to enhance the prediction accuracy of molecular properties. J Cheminform 15:54 37211605 10.1186/s13321-023-00712-0 PMC10200055

[50] Masisi L, Nelwamondo V, Marwala T (2008) The use of entropy to measure structural diversity. In: 2008 IEEE International Conference on Computational Cybernetics. IEEE

[51] Instale o TensorFlow 2. In: TensorFlow. https://www.tensorflow.org/install?hl=pt. Accessed 20 Sep 2024

[52] XGBoost Documentation — xgboost 2.1.1 documentation. https://xgboost.readthedocs.io/en/stable/. Accessed 20 Sep 2024

[53] Python-package introduction — LightGBM 4.0.0 documentation. https://lightgbm.readthedocs.io/en/stable/Python-Intro.html. Accessed 20 Sep 2024

[54] KNeighborsClassifier. In: scikit-learn. https://scikit-learn.org/dev/modules/generated/sklearn.neighbors.KNeighborsClassifier.html. Accessed 3 Oct 2024

[55] LogisticRegression. In: scikit-learn. https://scikit-learn.org/1.5/modules/generated/sklearn.linear_model.LogisticRegression.html. Accessed 3 Oct 2024

[56] RandomForestClassifier. In: scikit-learn. https://scikit-learn.org/stable/modules/generated/sklearn.ensemble.RandomForestClassifier.html. Accessed 20 Sep 2024

[57] StratifiedKFold. In: scikit-learn. https://scikit-learn.org/stable/modules/generated/sklearn.model_selection.StratifiedKFold.html. Accessed 20 Sep 2024

[58] Lipton ZC, Elkan C, Naryanaswamy B (2014) Optimal thresholding of classifiers to maximize F1 measure. Mach Learn Knowl Discov Databases 8725:225–239 26023687 10.1007/978-3-662-44851-9_15 PMC4442797

[59] Grandini M, Bagli E, Visani G (2020) Metrics for multi-class classification: An overview. arXiv [stat.ML]

