# Supplementary material for: GPCR-A17 MAAP: mapping modulators, agonists, and antagonists to predict the next bioactive target
Source: J Cheminform. 2025 Jul 11;17:102. doi: 10.1186/s13321-025-01050-z (PMC12255011; doi:10.1186/s13321-025-01050-z)
Supplement: Supplementary file 1 — Additional file 1. [file 13321_2025_1050_MOESM1_ESM.docx]

**Supplementary information**

**GPCR-A17 MAAP: Mapping modulators, agonists, and antagonists to Predict the Next Bioactive Target**

Ana B. Caniceiro^1,2,3,4^, Ana M. B. Amorim^1,2,3,4,5^, Nícia Rosário-Ferreira^1,2,4,5^, Irina S. Moreira^1,2,4,6*^

^1^CNC-UC - Center for Neuroscience and Cell Biology, University of Coimbra Portugal

^2^CiBB - Centre for Innovative Biomedicine and Biotechnology, University of Coimbra, Portugal.

^3^PhD in Biosciences, Department of Life Sciences, University of Coimbra, Calçada Martim de Freitas, 3000-456 Coimbra, Portugal

^4^Department of Life Sciences, University of Coimbra, Calçada Martim de Freitas, 3000-456 Coimbra, Portugal.

^5^PURR.AI, Rua Pedro Nunes, IPN Incubadora, Ed C, 3030-199 Coimbra, Portugal.

^6^Lead contact

*Correspondence: [irina.moreira@cnc.uc.pt](mailto:irina.moreira@cnc.uc.pt)

# **Supplementary Tables and Figures**

***Table S1******. Ranges of hyperparameters tuned and optimised hyperparameters for the RF, XGBoost, LightGBM, DNN, KNN, and LR models on the full dataset and Ki-filtered dataset using Optuna optimisation.*** *RF: Random Forest, XGBoost: Extreme Gradient Boosting, LightGBM: Light Gradient Boosting Machine, DNN: Deep Neural Network, KNN: K-Nearest Neighbours, LR: Logistic Regression.*

|  | ***Optimised Hyperparameter*** | ***Description*** | ***Range/Values*** | ***Full Dataset Value*** | ***Ki-Filtered Dataset*** |
| --- | --- | --- | --- | --- | --- |
| ***RF*** | *n_estimators* | *Number of trees (estimators)* | *100 to 1000* | *565* | *421* |
|  | *max_depth* | *Maximum tree depth* | *5 to 50* | *45* | *24* |
|  | *min_samples_split* | *Minimum number of samples required to split a node* | *2 to 10* | *2* | *6* |
|  | *min_samples_leaf* | *Minimum number of samples required in a leaf node* | *1 to 10* | *1* | *3* |
|  | *max_features* | *Maximum number of features considered for splitting* | *‘sqrt’, ‘log2’* | *‘sqrt’* | *‘sqrt’* |
|  | *boostrap* | *Whether bootstrap samples are used when building trees* | *True, False* | *False* | *False* |
|  | *criterion* | *Function to measure the quality of a split* | *‘gini’, ‘entropy’* | *‘entropy’* | *‘entropy’* |
| ***XGBoost*** | *n_estimators* | *Number of trees (estimators)* | *300 to 1000* | *978* | *572* |
|  | *learning_rate* | *Step size shrinkage* | *0.01 to 0.1* | *0.031787249844945395* | *0.027050495353217213* |
|  | *max_depth* | *Maximum tree depth* | *3 to 10* | *10* | *10* |
|  | *min_child_weight* | *Minimum sum of instance weights in a child node* | *3 to 20* | *3* | *5* |
|  | *gamma* | *Minimum loss reduction for partitioning* | *0.2 to 0.3* | *0.2196267504850338* | *0.2504415112409476* |
|  | *subsample* | *Fraction of samples per tree* | *0.6 to 0.9* | *0.8538685980954783* | *0.882178986350042* |
|  | *colsample_bytree* | *Fraction of features per tree* | *0.6 to 0.9* | *0.7405703449708528* | *0.800868163840265* |
|  | *reg_alpha* | *L1 regularisation term* | *0.1 to 4* | *0.5268967912463021* | *0.4212395635381482* |
|  | *reg_lambda* | *L2 regularisation term* | *1.5 to 5* | *3.332801089359329* | *4.349273356170861* |
| ***LightGBM*** | *n_estimators* | *Number of boosting rounds* | *300 to 1000* | *352* | *572* |
|  | *learning_rate* | *Step size shrinkage* | *0.01 to 0.1* | *0.07870311662571684* | *0.01597020644399367* |
|  | *max_depth* | *Maximum depth of each tree* | *3 to 10* | *10* | *6* |
|  | *min_child_weight* | *Minimum sum of instance weights in a child node* | *3 to 20* | *3* | *4* |
|  | *gamma* | *Minimum loss reduction required for further partitioning* | *0.2 to 0.3* | *0.2988838794671687* | *0.2820167302559291* |
|  | *subsample* | *Fraction of samples used per tree* | *0.7 to 0.9* | *0.8818476554804694* | *0.7625832298036728* |
|  | *colsample_bytree* | *Fraction of features used per tree* | *0.7 to 0.9* | *0.8872869255161334* | *0.7138923934210505* |
|  | *reg_alpha* | *L1 regularisation term on weights* | *0.1 to 4* | *0.10433653121552235* | *0.29198789947801423* |
|  | *reg_lambda* | *L2 regularisation term on weights* | *1.5 to 4* | *3.066768385314646* | *3.2982675160982717* |
| ***DNN*** | *n_layers* | *Number of layers in the neural network* | *1 to 7* | *2* | *1* |
|  | *nodes_per_layer* | *Number of neurons in each layer* | *8 to 64 (per layer)* | *'nodes_l0': 63; 'nodes_l1':21* | *'nodes_l0': 52* |
|  | *dropout_rate* | *Fraction of neurons dropped during training to prevent overfitting* | *0.1 to 0.5 (per layer)* | *'dropout_l0': 0.43618876067674395; 'dropout_l1':* *0.27139347017903864* | *'dropout_l0': 0.26092517809325466* |
|  | *l2_rate* | *L2 regularization coefficient to penalize large weights* | *0.00001 to 0.01 (per layer)* | *'l2_l0': 0.004655221285908134; 'l2_l1':* *0.005088265142703275* | *'l2_l0': 0.004259445714361405* |
|  | *learning_rate* | *Step size for updating weights* | *0.0001 to 0.01* | *0.00010933924632380652* | *0.0002714264131621993* |
|  | *batch_size* | *Number of samples processed before the model updates its weights during training* | *8, 16, 32, 64* | *64* | *32* |
|  | *epochs* | *Number of times the entire training dataset is passed forward and backward through the model* | *10 to 200* | *177* | *115* |
| ***KNN*** | *n_neighbors* | *Number of neighbours* | *3 to 15* | *3* | *3* |
|  | *weights* | *Weight function used in prediction* | *"uniform", "distance"* | *'distance'* | *‘distance'* |
|  | *algorithm* | *Algorithm used to compute nearest neighbours* | *"auto", "ball_tree", "kd_tree", "brute"* | *'ball_tree'* | *‘auto’* |
|  | *leaf_size* | *Size of the leaf in tree-based algorithms* | *20 to 50* | *20* | *21* |
|  | *p* | *Power parameter for the Minkowski distance metric* | *1 to 2* | *1* | *2* |
| ***LR*** | *C* | *Inverse of regularisation strength* | *0.01 to 10.0* | *7.447711624627201* | *5.561525079789082* |
|  | *max_iter* | *Maximum number of iterations for optimisation* | *100 to 1000* | *783* | *284* |
|  | *penalty* | *Type of regularisation applied* | *'l1', 'l2', 'elasticnet'* | *'l2'* | *'l1'* |
|  | *solver* | *Algorithm used for optimisation* | *'liblinear', 'saga'* | *'liblinear'* | *'liblinear'* |
|  | *l1_ratio* | *Only relevant when using 'elasticnet' as the penalty* | *-* | *-* | *-* |

***Table S2. F1 score (macro) of the methods on the internal validation set used for hyperparameter tuning.*** *The highest values are indicated in bold. GPCR: G Protein-Coupled Receptor, MAAP: modulator, agonist, antagonist predictor, DNN: Deep Neural Network, XGBoost: Extreme Gradient Boosting, LightGBM: Light Gradient-Boosting Machine, KNN: K-Nearest Neighbor, LR: Logistic Regression.*

| **Method** | **Best Validation F1 Score (macro) during Hyperparameter Tuning** |
| --- | --- |
| RF | 0.8701 |
| DNN | 0.8565 |
| XGBoost | **0.8792** |
| LightGBM | 0.8783 |
| KNN | 0.7665 |
| LR | 0.7989 |

***Table S3. DNN full-dataset classification report for each class in the independent ligand validation set.*** *Data are presented as the mean ± standard deviation. DNN: Deep Neural Network.*

| **Method** | **Dataset** | **Recall** | **Precision** | **F1 Score** | **F1 Number of Samples** |
| --- | --- | --- | --- | --- | --- |
| DNN | Antagonist | 0.8531 ± 0.0069 | *0.7183 ± 0.0087* | 0.7799 ± 0.0048 | 350 |
|  | Agonist | 0.6851 ± 0.0140 | 0.7207 ± 0.0089 | 0.7024 ± 0.0091 | 195 |
|  | Modulator | 0.3850 ± 0.0294 | 0.6234 ± 0.0113 | 0.4754 ± 0.0235 | 147 |

***Table S4. KNN full dataset classification report for each class in the independent ligand validation set****. Data are presented as the mean ± standard deviation. KNN: K-nearest neighbour.*

| **Method** | **Dataset** | **Recall** | **Precision** | **F1 Score** | **Number of Samples** |
| --- | --- | --- | --- | --- | --- |
| KNN | Antagonist | 0.8257 ± 0.0000 | 0.7189 ± 0.0000 | 0.7686 ± 0.0000 | 350 |
|  | Agonist | 0.7231 ± 0.0000 | 0.7015 ± 0.0000 | 0.7121 ± 0.0000 | 195 |
|  | Modulator | 0.3946 ± 0.0000 | 0.6517 ± 0.0000 | 0.4915 ± 0.0000 | 147 |

***Table S5. LR full dataset classification report for each class in the independent ligand validation set******.*** *Data are presented as the mean ± standard deviation. LR: Logistic Regression.*

| **Method** | **Dataset** | **Recall** | **Precision** | **F1 Score** | **Number of Samples** |
| --- | --- | --- | --- | --- | --- |
| LR | Antagonist | 0.7743 ± 0.0000 | 0.6985 ± 0.0008 | 0.7344 ± 0.0005 | 350 |
|  | Agonist | 0.6718 ± 0.0026 | 0.6179 ± 0.0022 | 0.6437 ± 0.0023 | 195 |
|  | Modulator | 0.2925 ± 0.0000 | 0.4674 ± 0.0032 | 0.3598 ± 0.0008 | 147 |

***Table S6. F1 score (macro) of the Ki-filtered models on the internal validation set used for hyperparameter tuning.*** *The highest values are highlighted in bold. RF: Random Forest, XGBoost: Extreme Gradient Boosting, LightGBM: Light Gradient-Boosting Machine, DNN: Deep Neural Network, LR: Logistic Regression, KNN: K-Nearest Neighbours.*

| **Method** | **Best Validation F1 Score (macro) during Hyperparameter Tuning** |
| --- | --- |
| RF | 0.9116 |
| XGBoost | 0.9192 |
| LightGBM | 0.9181 |
| DNN | **0.9282** |
| LR | 0.8712 |
| KNN | 0.8186 |

***Table S7. Performance of each Ki-filtered base method on the testing and independent ligand validation sets.*** *The best metrics are highlighted in bold for the testing set and in bold and underlined for the independent ligand validation set to distinguish the superior performance across both evaluation stages. Data are presented as the mean ± standard deviation. RF: Random Forest, DNN: Deep Neural Network, XGBoost: Extreme Gradient Boosting, LightGBM: Light Gradient Boosting Machine, KNN: K-Nearest Neighbours, LR: Logistic Regression.*

| **Dataset** | **Method** | **Recall** | **Precision** | **F1 Score** | **AUC** | **Specificity** |
| --- | --- | --- | --- | --- | --- | --- |
| Testing | RF (Ki-filtered) | 0.9158 ± 0.0021 | 0.9159 ± 0.0022 | 0.9153 ± 0.0021 | **0.9786 ± 0.0006** | 0.9694 ± 0.0008 |
| Independent  ligand validation |  | **0.8215 ± 0.0027** | **0.8205 ± 0.0027** | **0.8171 ± 0.0026** | **0.9022 ± 0.0011** | **0.9276 ± 0.0014** |
| Testing | DNN  (Ki-filtered) | 0.9075 ± 0.0035 | 0.9083 ± 0.0033 | 0.9076 ± 0.0036 | 0.9650 ± 0.0019 | 0.9660 ± 0.0014 |
| Independent  ligand validation |  | 0.7728 ± 0.0159 | 0.7816 ± 0.0112 | 0.7751 ± 0.0142 | 0.8591 ± 0.0084 | 0.9018 ± 0.0088 |
| Testing | XGBoost  (Ki-filtered) | 0.9268 ± 0.0010 | 0.9268 ± 0.0012 | 0.9268 ± 0.0011 | 0.9776 ± 0.0004 | 0.9737 ± 0.0004 |
| Independent  ligand validation |  | 0.8108 ± 0.0050 | 0.8095 ± 0.0051 | 0.8087 ± 0.0047 | 0.8970 ± 0.0014 | 0.9222 ± 0.0025 |
| Testing | LightGBM  (Ki-filtered) | **0.9278 ± 0.0010** | **0.9276 ± 0.0010** | **0 0.9277 ± 0.0010** | 0.9755 ± 0.0003 | **0.9741 ± 0.0004** |
| Independent  ligand validation |  | 0.8056 ± 0.0015 | 0.8043 ± 0.0015 | 0.8037 ± 0.0014 | 0.8973 ± 0.0016 | 0.9196 ± 0.0008 |
| Testing | KNN (Ki-filtered) | 0.8208 ± 0.0000 | 0.8196 ± 0.0000 | 0.8196 ± 0.0000 | 0.9025 ± 0.0000 | 0.9272 ± 0.0000 |
| Independent  ligand validation |  | 0.7611 ± 0.0000 | 0.7753 ± 0.0000 | 0.7657 ± 0.0000 | 0.8452 ± 0.0000 | 0.8954 ± 0.0000 |
| Testing | LR (Ki-filtered) | 0.8701 ± 0.0000 | 0.8689 ± 0.0000 | 0.8689 ± 0.0000 | 0.9294 ± 0.0001 | 0.9502 ± 0.0000 |
| Independent  ligand validation |  | 0.6796 ± 0.0009 | 0.6959 ± 0.0010 | 0.6857 ± 0.0009 | 0.8038 ± 0.0001 | 0.8429 ± 0.0007 |

***Table S8. RF, XGBoost, and LightGBM Ki-filtered base model classification reports for each class in the* *independent ligand validation set.*** *The best metrics for each class are highlighted in bold. Data are presented as the mean ± standard deviation. RF, Random Forest, XGBoost: Extreme Gradient Boosting, LightGBM: Light Gradient-Boosting Machine, Ki: Inhibition Constant.*

| **Dataset** | **Method** | **Recall** | **Precision** | **F1 Score** | **F1 Number of**  **Samples** |
| --- | --- | --- | --- | --- | --- |
| Antagonist | RF (Ki-filtered) | **0.9227 ± 0.0046** | **0.8364 ± 0.0043** | **0.8774 ± 0.0030** | 256 |
|  | XGBoost (Ki-filtered) | 0.8844 ± 0.0080 | 0.8336 ± 0.0018 | 0.8582 ± 0.0046 |  |
|  | LightGBM (Ki-filtered) | 0.8773 ± 0.0031 | 0.8319 ± 0.0022 | 0.8540 ± 0.0015 |  |
| Agonist | RF (Ki-filtered) | 0.6619 ± 0.0041 | **0.8403 ± 0.0046** | 0.7405 ± 0.0037 | 97 |
|  | XGBoost (Ki-filtered) | **0.6990 ± 0.0041** | 0.8092 ± 0.0122 | **0.7500 ± 0.0059** |  |
|  | LightGBM (Ki-filtered) | 0.6907 ± 0.0000 | 0.7957 ± 0.0038 | 0.7395 ± 0.0016 |  |
| Modulator | RF (Ki-filtered) | 0.6811 ± 0.0066 | **0.7395 ± 0.0157** | 0.7089 ± 0.0047 | 74 |
|  | XGBoost (Ki-filtered) | 0.7027 ± 0.0000 | 0.7264 ± 0.0104 | **0.7143 ± 0.0050** |  |
|  | LightGBM (Ki-filtered) | **0.7081 ± 0.0066** | 0.7199 ± 0.0084 | 0.7139 ± 0.0024 |  |

***Table S9. List of ligand descriptors from Mold2 used in GPCR-A17 MAAP and GPCR-A17 MAAP (Ki-filtered).*** *In the GPCR-A17 MAAP (Ki-filtered), the following descriptors were not used for model training or evaluation because of the data curation criteria: D033, D331, D369, D376, D387, D608, D637, D645, D646, D686, D693, D694, D701.*

| **Symbol** | **Definition** | **Symbol** | **Definition** |
| --- | --- | --- | --- |
| **D001** | Number of 6-membered aromatic rings (only carbon atoms) | **D413** | Walk-returning count order-5 |
| **D002** | Number of 03-membered rings | **D414** | Walk-returning count order-6 |
| **D003** | Number of 04-membered rings | **D415** | Topological structure autocorrelation length-1 weighted by atomic masses |
| **D004** | Number of 05-membered rings | **D416** | Topological structure autocorrelation length-2 weighted by atomic masses |
| **D005** | Number of 06-membered rings | **D417** | Topological structure autocorrelation length-3 weighted by atomic masses |
| **D006** | Number of 07-membered rings | **D418** | Topological structure autocorrelation length-4 weighted by atomic masses |
| **D007** | Number of 08-membered rings | **D419** | Topological structure autocorrelation length-5 weighted by atomic masses |
| **D009** | Number of 10-membered rings | **D420** | Topological structure autocorrelation length-6 weighted by atomic masses |
| **D010** | Number of 11-membered rings | **D421** | Topological structure autocorrelation length-7 weighted by atomic masses |
| **D011** | Number of 12-membered rings | **D422** | Topological structure autocorrelation length-8 weighted by atomic masses |
| **D012** | Number of multiple bonds | **D423** | Topological structure autocorrelation length-1 weighted by atomic van der Waals volumes |
| **D013** | Number of circuits structure | **D424** | Topological structure autocorrelation length-2 weighted by atomic van der Waals volumes |
| **D014** | Number of rotatable bonds | **D425** | Topological structure autocorrelation length-3 weighted by atomic van der Waals volumes |
| **D015** | Rotatable bond fraction | **D426** | Topological structure autocorrelation length-4 weighted by atomic van der Waals volumes |
| **D016** | Number of double bonds | **D427** | Topological structure autocorrelation length-5 weighted by atomic van der Waals volumes |
| **D017** | Number of aromatic bonds | **D428** | Topological structure autocorrelation length-6 weighted by atomic van der Waals volumes |
| **D018** | Sum of conventional bond orders (H-depleted) | **D429** | Topological structure autocorrelation length-7 weighted by atomic van der Waals volumes |
| **D019** | Number of hydrogen | **D430** | Topological structure autocorrelation length-8 weighted by atomic van der Waals volumes |
| **D023** | Number of boron | **D431** | Topological structure autocorrelation length-1 weighted by atomic Sanderson electronegativities |
| **D024** | Number of carbon | **D432** | Topological structure autocorrelation length-2 weighted by atomic Sanderson electronegativities |
| **D025** | Number of nitrogen | **D433** | Topological structure autocorrelation length-3 weighted by atomic Sanderson electronegativities |
| **D026** | Number of oxygen | **D434** | Topological structure autocorrelation length-4 weighted by atomic Sanderson electronegativities |
| **D027** | Number of fluorine | **D435** | Topological structure autocorrelation length-5 weighted by atomic Sanderson electronegativities |
| **D032** | Number of silicon | **D436** | Topological structure autocorrelation length-6 weighted by atomic Sanderson electronegativities |
| **D033** | Number of phosphorus | **D437** | Topological structure autocorrelation length-7 weighted by atomic Sanderson electronegativities |
| **D034** | Number of sulfur | **D438** | Topological structure autocorrelation length-8 weighted by atomic Sanderson electronegativities |
| **D035** | Number of chlorine | **D439** | Topological structure autocorrelation length-1 weighted by atomic polarizabilities |
| **D052** | Number of selenium | **D440** | Topological structure autocorrelation length-2 weighted by atomic polarizabilities |
| **D053** | Number of bromine | **D441** | Topological structure autocorrelation length-3 weighted by atomic polarizabilities |
| **D071** | Number of iodine | **D442** | Topological structure autocorrelation length-4 weighted by atomic polarizabilities |
| **D122** | Molecular weight | **D443** | Topological structure autocorrelation length-5 weighted by atomic polarizabilities |
| **D123** | Average of molecular weight | **D444** | Topological structure autocorrelation length-6 weighted by atomic polarizabilities |
| **D124** | Number of atoms in each molecule | **D445** | Topological structure autocorrelation length-7 weighted by atomic polarizabilities |
| **D125** | Number of none-hydrogen atoms in each molecule | **D446** | Topological structure autocorrelation length-8 weighted by atomic polarizabilities |
| **D126** | Number of bonds in each molecule | **D447** | Geary topological structure autocorrelation length-1 weighted by atomic masses |
| **D127** | Number of none-hydrogen bonds in each molecule | **D448** | Geary topological structure autocorrelation length-2 weighted by atomic masses |
| **D128** | Number of rings in each molecule | **D449** | Geary topological structure autocorrelation length-3 weighted by atomic masses |
| **D129** | Number of triple bonds in each molecule | **D450** | Geary topological structure autocorrelation length-4 weighted by atomic masses |
| **D130** | Number of halogen atoms in each molecule | **D451** | Geary topological structure autocorrelation length-5 weighted by atomic masses |
| **D131** | Molecular size index | **D452** | Geary topological structure autocorrelation length-6 weighted by atomic masses |
| **D132** | Atomic composition index | **D453** | Geary topological structure autocorrelation length-7 weighted by atomic masses |
| **D133** | Mean value of atomic composition index | **D454** | Geary topological structure autocorrelation length-8 weighted by atomic masses |
| **D134** | Branch index | **D455** | Geary topological structure autocorrelation length-1 weighted by atomic van der Waals volumes |
| **D135** | Molecular structure connectivity index | **D456** | Geary topological structure autocorrelation length-2 weighted by atomic van der Waals volumes |
| **D136** | Narumi-type topological index | **D457** | Geary topological structure autocorrelation length-3 weighted by atomic van der Waals volumes |
| **D137** | Harmonic topological index | **D458** | Geary topological structure autocorrelation length-4 weighted by atomic van der Waals volumes |
| **D138** | Geometric topological index | **D459** | Geary topological structure autocorrelation length-5 weighted by atomic van der Waals volumes |
| **D139** | Topological distance count order-3 | **D460** | Geary topological structure autocorrelation length-6 weighted by atomic van der Waals volumes |
| **D140** | Log of vertex distance path count | **D461** | Geary topological structure autocorrelation length-7 weighted by atomic van der Waals volumes |
| **D141** | Average of vertex distance path count | **D462** | Geary topological structure autocorrelation length-8 weighted by atomic van der Waals volumes |
| **D142** | Balaban type of mean square vertex distance index | **D463** | Geary topological structure autocorrelation length-1 weighted by atomic Sanderson electronegativities |
| **D143** | Sum of atomic van der Waals carbon-scale | **D464** | Geary topological structure autocorrelation length-2 weighted by atomic Sanderson electronegativities |
| **D144** | Mean atomic van der Waals carbon-scale | **D465** | Geary topological structure autocorrelation length-3 weighted by atomic Sanderson electronegativities |
| **D145** | Sum of atomic electronegativities Pauling-scale on carbon | **D466** | Geary topological structure autocorrelation length-4 weighted by atomic Sanderson electronegativities |
| **D146** | Mean atomic electronegativities Pauling-scaled on carbon | **D467** | Geary topological structure autocorrelation length-5 weighted by atomic Sanderson electronegativities |
| **D147** | Sum of atomic electronegativities Sanderson-scaled on carbon | **D468** | Geary topological structure autocorrelation length-6 weighted by atomic Sanderson electronegativities |
| **D148** | Mean atomic electronegativity Sanderson-scaled on carbon | **D469** | Geary topological structure autocorrelation length-7 weighted by atomic Sanderson electronegativities |
| **D149** | Sum of atomic electronegativities Allred-Rochow-scaled on carbon | **D470** | Geary topological structure autocorrelation length-8 weighted by atomic Sanderson electronegativities |
| **D150** | Mean atomic electronegativity Allred-Rochow-scaled on carbon | **D471** | Geary topological structure autocorrelation length-1 weighted by atomic polarizabilities |
| **D151** | Sum of atomic polarizabilities scaled on carbon-SP3 | **D472** | Geary topological structure autocorrelation length-2 weighted by atomic polarizabilities |
| **D152** | Mean atomic polarizability scaled on carbon-SP3 | **D473** | Geary topological structure autocorrelation length-3 weighted by atomic polarizabilities |
| **D153** | Zagreb order-1 index | **D474** | Geary topological structure autocorrelation length-4 weighted by atomic polarizabilities |
| **D154** | Zagreb order-1 index with value of valence vertex degrees | **D475** | Geary topological structure autocorrelation length-5 weighted by atomic polarizabilities |
| **D155** | Zagreb order-2 index | **D476** | Geary topological structure autocorrelation length-6 weighted by atomic polarizabilities |
| **D156** | Vertex degree topological index | **D477** | Geary topological structure autocorrelation length-7 weighted by atomic polarizabilities |
| **D157** | Second Zagreb order-2 index with value of valence vertex degrees | **D478** | Geary topological structure autocorrelation length-8 weighted by atomic polarizabilities |
| **D158** | Valence electrons of principal quantum index | **D479** | Moran topological structure autocorrelation length-1 weighted by atomic masses |
| **D159** | Schultz type molecular topological index | **D480** | Moran topological structure autocorrelation length-2 weighted by atomic masses |
| **D160** | Schultz type molecular topological index of valence vertex degrees | **D481** | Moran topological structure autocorrelation length-3 weighted by atomic masses |
| **D161** | Molecular topological distance index | **D482** | Moran topological structure autocorrelation length-4 weighted by atomic masses |
| **D162** | Molecular topological distance index of valence vertex degrees | **D483** | Moran topological structure autocorrelation length-5 weighted by atomic masses |
| **D163** | Molecular size and branching index | **D484** | Moran topological structure autocorrelation length-6 weighted by atomic masses |
| **D164** | Index of terminal vertex matrix | **D485** | Moran topological structure autocorrelation length-7 weighted by atomic masses |
| **D165** | Wiener index | **D486** | Moran topological structure autocorrelation length-8 weighted by atomic masses |
| **D166** | Average path length in Wiener Index | **D487** | Moran topological structure autocorrelation length-1 weighted by atomic van der Waals volumes |
| **D167** | Reciprocal index of Wiener distance matrix | **D488** | Moran topological structure autocorrelation length-2 weighted by atomic van der Waals volumes |
| **D168** | Harary index | **D489** | Moran topological structure autocorrelation length-3 weighted by atomic van der Waals volumes |
| **D169** | Index of Laplacian matrix | **D490** | Moran topological structure autocorrelation length-4 weighted by atomic van der Waals volumes |
| **D170** | First non-zero eigenvalue of Laplacian matrix | **D491** | Moran topological structure autocorrelation length-5 weighted by atomic van der Waals volumes |
| **D171** | Wiener–path index | **D492** | Moran topological structure autocorrelation length-6 weighted by atomic van der Waals volumes |
| **D172** | Reciprocal Wiener-path index | **D493** | Moran topological structure autocorrelation length-7 weighted by atomic van der Waals volumes |
| **D173** | Mohar order-2 index | **D494** | Moran topological structure autocorrelation length-8 weighted by atomic van der Waals volumes |
| **D174** | Maximum path index | **D495** | Moran topological structure autocorrelation length-1 weighted by atomic Sanderson electronegativities |
| **D175** | Wiener type maximum path index | **D496** | Moran topological structure autocorrelation length-2 weighted by atomic Sanderson electronegativities |
| **D176** | Reciprocal Wiener type maximum path index | **D497** | Moran topological structure autocorrelation length-3 weighted by atomic Sanderson electronegativities |
| **D177** | Minimum-path/maximum-path index | **D498** | Moran topological structure autocorrelation length-4 weighted by atomic Sanderson electronegativities |
| **D178** | All-path Wiener - sum of the edges in the shortest paths between all pairs of non-hydrogen atoms | **D499** | Moran topological structure autocorrelation length-5 weighted by atomic Sanderson electronegativities |
| **D179** | Heteroatoms and multiple bonds weighted distance matrix | **D500** | Moran topological structure autocorrelation length-6 weighted by atomic Sanderson electronegativities |
| **D180** | Mass weighted distance matrix | **D501** | Moran topological structure autocorrelation length-7 weighted by atomic Sanderson electronegativities |
| **D181** | Index of van der Waals weighted distance matrix | **D502** | Moran topological structure autocorrelation length-8 weighted by atomic Sanderson electronegativities |
| **D182** | Distance matrix of electronegativity weighted with electronegativities Pauling-scale | **D503** | Moran topological structure autocorrelation length-1 weighted by atomic polarizabilities |
| **D183** | Distance matrix of electronegativity weighted with Sanderson electronegativities | **D504** | Moran topological structure autocorrelation length-2 weighted by atomic polarizabilities |
| **D184** | Distance matrix of electronegativity weighted with Allred-Rochow electronegativites | **D505** | Moran topological structure autocorrelation length-3 weighted by atomic polarizabilities |
| **D185** | Polarizability weighted distance matrix | **D506** | Moran topological structure autocorrelation length-4 weighted by atomic polarizabilities |
| **D186** | Average vertex distance connectivity index | **D507** | Moran topological structure autocorrelation length-5 weighted by atomic polarizabilities |
| **D187** | Balaban heteroatoms bonds weighted index | **D508** | Moran topological structure autocorrelation length-6 weighted by atomic polarizabilities |
| **D188** | Balaban mass weighted index | **D509** | Moran topological structure autocorrelation length-7 weighted by atomic polarizabilities |
| **D189** | Balaban van der Waals weighted index | **D510** | Moran topological structure autocorrelation length-8 weighted by atomic polarizabilities |
| **D190** | Balaban electronegativity weighted with Pauling-scale index | **D511** | Molecular topological order-1 charge index |
| **D191** | Balaban electronegativity weighted with Sanderson-scale index | **D512** | Molecular topological order-2 charge index |
| **D192** | Balaban electronegativity weighted with Allred-Rochow-scale index | **D513** | Molecular topological order-3 charge index |
| **D193** | Balaban-type polarizability weighted index | **D514** | Molecular topological order-4 charge index |
| **D194** | Maximal valence vertex electrotopological negative variation | **D515** | Molecular topological order-5 charge index |
| **D195** | Maximal valence vertex electrotopological positive variation | **D516** | Molecular topological order-6 charge index |
| **D196** | Sum absolute electrotopological negative variation | **D517** | Molecular topological order-7 charge index |
| **D197** | Electrotopological index | **D518** | Molecular topological order-8 charge index |
| **D198** | Sum electrotopological states index | **D519** | Molecular topological order-9 charge index |
| **D199** | Mean electrotopological states index | **D520** | Molecular topological order-10 charge index |
| **D200** | Vertex connectivity order-0 index | **D521** | Mean molecular topological order-1 charge index |
| **D201** | Vertex connectivity order-1 index | **D522** | Mean molecular topological order-2 charge index |
| **D202** | Vertex connectivity order-2 index | **D523** | Mean molecular topological order-3 charge index |
| **D203** | Vertex connectivity order-3 index | **D524** | Mean molecular topological order-4 charge index |
| **D204** | Vertex connectivity order-4 index | **D525** | Mean molecular topological order-5 charge index |
| **D205** | Vertex connectivity order-5 index | **D526** | Mean molecular topological order-6 charge index |
| **D206** | Average vertex connectivity order-0 index | **D527** | Mean molecular topological order-7 charge index |
| **D207** | Average vertex connectivity order-1 index | **D528** | Mean molecular topological order-8 charge index |
| **D208** | Average vertex connectivity order-2 index | **D529** | Mean molecular topological order-9 charge index |
| **D209** | Average vertex connectivity order-3 index | **D530** | Mean molecular topological order-10 charge index |
| **D210** | Average vertex connectivity order-4 index | **D531** | Sum of molecular topological mean charge index |
| **D211** | Average vertex connectivity order-5 index | **D532** | Lowest eigenvalue from Burdex matrix weighted by masses order-1 |
| **D212** | Valence vertex connectivity order-0 index | **D533** | Lowest eigenvalue from Burdex matrix weighted by masses order-2 |
| **D213** | Valence vertex connectivity order-1 index | **D534** | Lowest eigenvalue from Burdex matrix weighted by masses order-3 |
| **D214** | Valence vertex connectivity order-2 index | **D535** | Lowest eigenvalue from Burdex matrix weighted by masses order-4 |
| **D215** | Valence vertex connectivity order-3 index | **D536** | Lowest eigenvalue from Burdex matrix weighted by masses order-5 |
| **D216** | Valence vertex connectivity order-4 index | **D537** | Lowest eigenvalue from Burdex matrix weighted by masses order-6 |
| **D217** | Valence vertex connectivity order-5 index | **D538** | Lowest eigenvalue from Burdex matrix weighted by masses order-7 |
| **D218** | Average valence vertex connectivity order-0 index | **D539** | Lowest eigenvalue from Burdex matrix weighted by masses order-8 |
| **D219** | Average valence vertex connectivity order-1 index | **D540** | Lowest eigenvalue from Burdex matrix weighted by van der Waals order-1 |
| **D220** | Average valence vertex connectivity order-2 index | **D541** | Lowest eigenvalue from Burdex matrix weighted by van der Waals order-2 |
| **D221** | Average valence vertex connectivity order-3 index | **D542** | Lowest eigenvalue from Burdex matrix weighted by van der Waals order-3 |
| **D222** | Average valence vertex connectivity order-4 index | **D543** | Lowest eigenvalue from Burdex matrix weighted by van der Waals order-4 |
| **D223** | Average valence vertex connectivity order-5 index | **D544** | Lowest eigenvalue from Burdex matrix weighted by van der Waals order-5 |
| **D224** | Principal quantum vertex connectivity order-0 index | **D545** | Lowest eigenvalue from Burdex matrix weighted by van der Waals order-6 |
| **D225** | Principal quantum vertex connectivity order-1 index | **D546** | Lowest eigenvalue from Burdex matrix weighted by van der Waals order-7 |
| **D226** | Principal quantum vertex connectivity order-2 index | **D547** | Lowest eigenvalue from Burdex matrix weighted by van der Waals order-8 |
| **D227** | Principal quantum vertex connectivity order-3 index | **D548** | Lowest eigenvalue from Burdex matrix weighted by electronegativities Sanderson-Scale order-1 |
| **D228** | Principal quantum vertex connectivity order-4 index | **D549** | Lowest eigenvalue from Burdex matrix weighted by electronegativities Sanderson-Scale order-2 |
| **D229** | Principal quantum vertex connectivity order-5 index | **D550** | Lowest eigenvalue from Burdex matrix weighted by electronegativities Sanderson-Scale order-3 |
| **D230** | Aromaticity valence vertex connectivity order-1 index | **D551** | Lowest eigenvalue from Burdex matrix weighted by electronegativities Sanderson-Scale order-4 |
| **D231** | Sum of valence vertex connectivity order-1 index | **D552** | Lowest eigenvalue from Burdex matrix weighted by electronegativities Sanderson-Scale order-5 |
| **D232** | Reciprocal distance order-1 sum product index | **D553** | Lowest eigenvalue from Burdex matrix weighted by electronegativities Sanderson-Scale order-6 |
| **D233** | Squared reciprocal distance order-1 sum product index | **D554** | Lowest eigenvalue from Burdex matrix weighted by electronegativities Sanderson-Scale order-7 |
| **D234** | Kier atom's 0-order path information index | **D555** | Lowest eigenvalue from Burdex matrix weighted by electronegativities Sanderson-Scale order-8 |
| **D235** | Kier 1-path index | **D556** | Lowest eigenvalue from Burdex matrix weighted by polarizabilities order-1 |
| **D236** | Kier 2-path index | **D557** | Lowest eigenvalue from Burdex matrix weighted by polarizabilities order-2 |
| **D237** | Kier 3-path index | **D558** | Lowest eigenvalue from Burdex matrix weighted by polarizabilities order-3 |
| **D238** | Molecular flexibility index | **D559** | Lowest eigenvalue from Burdex matrix weighted by polarizabilities order-4 |
| **D239** | Atom's connectivity index in longest path | **D560** | Lowest eigenvalue from Burdex matrix weighted by polarizabilities order-5 |
| **D240** | Sum of the longest path of the atom | **D561** | Lowest eigenvalue from Burdex matrix weighted by polarizabilities order-6 |
| **D241** | Average longest path of the molecule | **D562** | Lowest eigenvalue from Burdex matrix weighted by polarizabilities order-7 |
| **D242** | Average of deviation of average of longest path | **D563** | Lowest eigenvalue from Burdex matrix weighted by polarizabilities order-8 |
| **D243** | Average of deviation of distance degree | **D564** | Highest eigenvalue from Burdex matrix weighted by masses order-1 |
| **D244** | Shortest path in the molecule | **D565** | Highest eigenvalue from Burdex matrix weighted by masses order-2 |
| **D245** | Shortest path centralization index | **D566** | Highest eigenvalue from Burdex matrix weighted by masses order-3 |
| **D246** | Maximum value of variation | **D567** | Highest eigenvalue from Burdex matrix weighted by masses order-4 |
| **D247** | EXP2 of path-distance / walk-distance over all atoms | **D568** | Highest eigenvalue from Burdex matrix weighted by masses order-5 |
| **D248** | EXP3 of path-distance / walk-distance over all atoms | **D569** | Highest eigenvalue from Burdex matrix weighted by masses order-6 |
| **D249** | EXP4 of path-distance / walk-distance over all atoms | **D570** | Highest eigenvalue from Burdex matrix weighted by masses order-7 |
| **D250** | EXP5 of path-distance / walk-distance over all atoms | **D571** | Highest eigenvalue from Burdex matrix weighted by masses order-8 |
| **D251** | Petitjean index | **D572** | Highest eigenvalue from Burdex matrix weighted by van der Waals order-1 |
| **D252** | Structure centric index | **D573** | Highest eigenvalue from Burdex matrix weighted by van der Waals order-2 |
| **D253** | Structure looping centric group index | **D574** | Highest eigenvalue from Burdex matrix weighted by van der Waals order-3 |
| **D254** | Radial centric index | **D575** | Highest eigenvalue from Burdex matrix weighted by van der Waals order-4 |
| **D255** | Vertex distance count equality index | **D576** | Highest eigenvalue from Burdex matrix weighted by van der Waals order-5 |
| **D256** | Vertex distance count magnitude index | **D577** | Highest eigenvalue from Burdex matrix weighted by van der Waals order-6 |
| **D257** | Total vertex distance count equality index | **D578** | Highest eigenvalue from Burdex matrix weighted by van der Waals order-7 |
| **D258** | Total vertex distance count magnitude index | **D579** | Highest eigenvalue from Burdex matrix weighted by van der Waals order-8 |
| **D259** | Mean of distance degree equality index | **D580** | Highest eigenvalue from Burdex matrix weighted by electronegativities Sanderson-scale order-1 |
| **D260** | Mean of distance degree magnitude index | **D581** | Highest eigenvalue from Burdex matrix weighted by electronegativities Sanderson-scale order-2 |
| **D261** | Information of vertex degree equality index | **D582** | Highest eigenvalue from Burdex matrix weighted by electronegativities Sanderson-scale order-3 |
| **D262** | Information of bonds index | **D583** | Highest eigenvalue from Burdex matrix weighted by electronegativities Sanderson-scale order-4 |
| **D263** | Vertex distance path count index | **D584** | Highest eigenvalue from Burdex matrix weighted by electronegativities Sanderson-scale order-5 |
| **D264** | Complexity vertex distance path count index | **D585** | Highest eigenvalue from Burdex matrix weighted by electronegativities Sanderson-scale order-6 |
| **D265** | Vertex distance information index | **D586** | Highest eigenvalue from Burdex matrix weighted by electronegativities Sanderson-scale order-7 |
| **D266** | Relative of vertex distance information index | **D587** | Highest eigenvalue from Burdex matrix weighted by electronegativities Sanderson-scale order-8 |
| **D267** | Mean of vertex distance information index | **D588** | Highest eigenvalue from Burdex matrix weighted by polarizabilities order-1 |
| **D268** | Extended of vertex distance information index | **D589** | Highest eigenvalue from Burdex matrix weighted by polarizabilities order-2 |
| **D269** | Information content order-0 index | **D590** | Highest eigenvalue from Burdex matrix weighted by polarizabilities order-3 |
| **D270** | Information content order-1 index | **D591** | Highest eigenvalue from Burdex matrix weighted by polarizabilities order-4 |
| **D271** | Information content order-2 index | **D592** | Highest eigenvalue from Burdex matrix weighted by polarizabilities order-5 |
| **D272** | Information content order-3 index | **D593** | Highest eigenvalue from Burdex matrix weighted by polarizabilities order-6 |
| **D273** | Information content order-4 index | **D594** | Highest eigenvalue from Burdex matrix weighted by polarizabilities order-7 |
| **D274** | Information content order-5 index | **D595** | Highest eigenvalue from Burdex matrix weighted by polarizabilities order-8 |
| **D275** | Total information content order-0 index | **D596** | Number of total primary C-sp3 |
| **D276** | Total information content order-1 index | **D597** | Number of total secondary C-sp3 |
| **D277** | Total information content order-2 index | **D598** | Number of total tertiary C-sp3 |
| **D278** | Total information content order-3 index | **D599** | Number of total quaternary C-sp3 |
| **D279** | Total information content order-4 index | **D600** | Number of ring secondary C-sp3 |
| **D280** | Total information content order-5 index | **D601** | Number of ring tertiary C-sp3 |
| **D281** | Structural information content order-0 index | **D602** | Number of ring quaternary C-sp3 |
| **D282** | Structural information content order-1 index | **D603** | Number of unsubstituted aromatic C-sp2 |
| **D283** | Structural information content order-2 index | **D604** | Number of substituted aromatic C-sp2 |
| **D284** | Structural information content order-3 index | **D605** | Number of primary C-sp2 |
| **D285** | Structural information content order-4 index | **D606** | Number of secondary C-sp2 |
| **D286** | Structural information content order-5 index | **D607** | Number of tertiary C-sp2 |
| **D287** | Complementary information content order-0 index | **D608** | Number of group allenes |
| **D288** | Complementary information content order-1 index | **D609** | Number of terminal C-sp |
| **D289** | Complementary information content order-2 index | **D610** | Number of non-terminal C-sp |
| **D290** | Complementary information content order-3 index | **D619** | Number of group carboxylic acids (aliphatic) |
| **D291** | Complementary information content order-4 index | **D620** | Number of group carboxylic acids (aromatic) |
| **D292** | Complementary information content order-5 index | **D621** | Number of group esters (aliphatic) |
| **D293** | Bond information content order-0 index | **D622** | Number of group esters (aromatic) |
| **D294** | Bond information content order-1 index | **D623** | Number of group primary amides (aliphatic) |
| **D295** | Bond information content order-2 index | **D624** | Number of group primary amides (aromatic) |
| **D296** | Bond information content order-3 index | **D625** | Number of group secondary amides (aliphatic) |
| **D297** | Bond information content order-4 index | **D626** | Number of group secondary amides (aromatic) |
| **D298** | Bond information content order-5 index | **D627** | Number of group tertiary amides (aliphatic) |
| **D299** | The largest eigenvalue | **D628** | Number of group tertiary amides (aromatic) |
| **D300** | Spanning tree with log value | **D629** | Number of group carbamates (aliphatic) |
| **D301** | Maximum eigenvalue weighted by heteroatoms and multiple bonds matrix | **D630** | Number of group carbamates (aromatic) |
| **D302** | Maximum eigenvalue weighted by mass distance matrix | **D637** | Number of group thioesters (aliphatic) |
| **D303** | Maximum eigenvalue weighted by van der Waals distance matrix | **D642** | Number of group aldehydes (aromatic) |
| **D304** | Maximum eigenvalue weighted by polarizability distance matrix | **D643** | Number of group ketones (aliphatic) |
| **D305** | Maximum eigenvalue weighted by electronegativity Pauling-scale distance matrix | **D644** | Number of group ketones (aromatic) |
| **D306** | Maximum eigenvalue weighted by electronegativity Sanderson-scale weighted distance matrix | **D645** | Number of group urea derivatives |
| **D307** | Maximum eigenvalue weighted by electronegativity Allred-Rochow-scale distance matrix | **D646** | Number of group urea derivatives (aromatic) |
| **D308** | Sum eigenvalue weighted by heteroatoms and multiple bonds distance matrix | **D647** | Number of group primary amines (aliphatic) |
| **D309** | Sum eigenvalue weighted by mass distance matrix | **D648** | Number of group primary amines (aromatic) |
| **D310** | Sum eigenvalue weighted by van der Waals distance matrix | **D649** | Number of group secondary amines (aliphatic) |
| **D311** | Sum eigenvalue weighted by polarizability distance matrix | **D650** | Number of group secondary amines (aromatic) |
| **D312** | Sum eigenvalue weighted by electronegativity Pauling-scale distance matrix | **D651** | Number of group tertiary amines (aliphatic) |
| **D313** | Sum eigenvalue weighted by electronegativity Sanderson-scale distance matrix | **D652** | Number of group tertiary amines (aromatic) |
| **D314** | Sum eigenvalue weighted by electronegativity Allred-Rochow-scale distance matrix | **D657** | Number of group nitriles (aliphatic) |
| **D315** | Sum absolute eigenvalue weighted by heteroatoms and multiple bonds distance matrix | **D658** | Number of group nitriles (aromatic) |
| **D316** | Sum absolute eigenvalue weighted by mass distance matrix | **D667** | Number of group N-nitroso (aliphatic) |
| **D317** | Sum absolute eigenvalue weighted by van der Waals distance matrix | **D668** | Number of group N-nitroso (aromatic) |
| **D318** | Sum absolute eigenvalue weighted by polarizability distance matrix | **D669** | Number of group nitroso (aliphatic) |
| **D319** | Sum absolute eigenvalue weighted by electronegativity Pauling-scale distance matrix | **D670** | Number of group nitroso (aromatic) |
| **D320** | Sum absolute eigenvalue weighted by electronegativity Sanderson-scale distance matrix | **D671** | Number of group nitro (aliphatic) |
| **D321** | Sum absolute eigenvalue weighted by electronegativity Allred-Rochow-scale distance matrix | **D672** | Number of group nitro (aromatic) |
| **D322** | Distance+detour path with ring index of order 3 | **D674** | Number of group total hydroxyl groups |
| **D323** | Distance+detour path with ring index of order 4 | **D675** | Number of group phenols |
| **D324** | Distance+detour path with ring index of order 5 | **D676** | Number of group primary alcohols (aliphatic) |
| **D325** | Distance+detour path with ring index of order 6 | **D677** | Number of group secondary alcohols (aliphatic) |
| **D326** | Distance+detour path with ring index of order 7 | **D678** | Number of group tertiary alcohols (aliphatic) |
| **D328** | Distance+detour path with ring index of order 9 | **D679** | Number of group ethers (aliphatic) |
| **D329** | Distance+detour path with ring index of order 10 | **D680** | Number of group ethers (aromatic) |
| **D330** | Distance+detour path with ring index of order 11 | **D684** | Number of group sulfoxides |
| **D331** | Distance+detour path with ring index of order 12 | **D686** | Number of group sulfates |
| **D332** | Distance+detour path on ring index of order 3 (circuits) | **D689** | Number of group solfures |
| **D333** | Distance+detour path on ring index of order 4 (circuits) | **D692** | Number of group sulfonamides |
| **D334** | Distance+detour path on ring index of order 5 (circuits) | **D693** | Number of group phosphites |
| **D335** | Distance+detour path on ring index of order 6 (circuits) | **D694** | Number of group phosphates |
| **D336** | Distance+detour path on ring index of order 7 (circuits) | **D701** | Number of group R=CHX |
| **D337** | Distance+detour path on ring index of order 8 (circuits) | **D703** | Number of group R#CX |
| **D338** | Distance+detour path on ring index of order 9 (circuits) | **D708** | Number of group X-C on aromatic ring |
| **D339** | Distance+detour path on ring index of order 10 (circuits) | **D709** | Number of group X-C- on ring |
| **D340** | Distance+detour path on ring index of order 11 (circuits) | **D710** | Number of group X-C= on ring |
| **D341** | Distance+detour path on ring index of order 12 (circuits) | **D711** | Number of group X-C on conjugated C |
| **D342** | Molecular topological path index of order 02 | **D712** | Number of group donor atoms for H-bonds (with N and O) |
| **D343** | Molecular topological path index of order 03 | **D713** | Number of group acceptor atoms for H-bonds (N O F) |
| **D344** | Molecular topological path index of order 04 | **D714** | Number of group CH3R and CH4 |
| **D345** | Molecular topological path index of order 05 | **D715** | Number of group CH2R2 |
| **D346** | Molecular topological path index of order 06 | **D716** | Number of group CHR3 |
| **D347** | Molecular topological path index of order 07 | **D717** | Number of group CR4 |
| **D348** | Molecular topological path index of order 08 | **D718** | Number of group CH3X |
| **D349** | Molecular topological path index of order 09 | **D719** | Number of group CH2RX |
| **D350** | Molecular topological path index of order 10 | **D720** | Number of group CH2X2 |
| **D351** | Molecular topological multiple path index of order 03 | **D721** | Number of group CHR2X |
| **D352** | Molecular topological multiple path index of order 04 | **D722** | Number of group CHRX2 |
| **D353** | Molecular topological multiple path index of order 05 | **D723** | Number of group CHX3 |
| **D354** | Molecular topological multiple path index of order 06 | **D724** | Number of group CR3X |
| **D355** | Molecular topological multiple path index of order 07 | **D725** | Number of group CR2X2 |
| **D356** | Molecular topological multiple path index of order 08 | **D726** | Number of group CRX3 |
| **D357** | Molecular topological multiple path index of order 09 | **D727** | Number of group CX4 |
| **D358** | Molecular topological multiple path index of order 10 | **D728** | Number of group =CH2 |
| **D359** | Molecular topological all path index | **D729** | Number of group =CHR |
| **D360** | Conventional bond index | **D730** | Number of group =CR2 |
| **D361** | Ratio of convention bonds with total path counts | **D731** | Number of group =CHX |
| **D362** | Ratio of difference of conventional bonds and total path counts | **D732** | Number of group =CRX |
| **D363** | Randic index | **D733** | Number of group =CX2 |
| **D364** | Balaban all-path index | **D734** | Number of group #CH |
| **D365** | Balaban short-path index | **D735** | Number of group #CR or R=C=R |
| **D366** | Sum of topological distance between the vertices N and N | **D736** | Number of group #CX |
| **D367** | Sum of topological distance between the vertices N and P | **D737** | Number of group R~CH~R |
| **D368** | Sum of topological distance between the vertices N and O | **D738** | Number of group R~CR~R |
| **D369** | Sum of topological distance between the vertices N and S | **D739** | Number of group R~CX~R |
| **D370** | Sum of topological distance between the vertices N and F | **D740** | Number of group Al-CH=X |
| **D371** | Sum of topological distance between the vertices N and Cl | **D741** | Number of group Ar-CH=X |
| **D372** | Sum of topological distance between the vertices N and Br | **D742** | Number of group Al-C(=X)-Al |
| **D373** | Sum of topological distance between the vertices N and I | **D743** | Number of group Ar-C(=X)-R |
| **D374** | Sum of topological distance between the vertices O and O | **D744** | Number of group R-C(=X)-X / R-C#X |
| **D375** | Sum of topological distance between the vertices O and S | **D745** | Number of group X-C(=X)-X |
| **D376** | Sum of topological distance between the vertices O and P | **D746** | Number of group H attached to C0(sp3) no X attached to next C |
| **D377** | Sum of topological distance between the vertices O and F | **D747** | Number of group H attached to heteroatom |
| **D378** | Sum of topological distance between the vertices O and Cl | **D748** | Number of group H attached to C0(sp3) with 1X attached to next C |
| **D379** | Sum of topological distance between the vertices O and Br | **D749** | Number of group H attached to C0(sp3) with 2X attached to next C |
| **D380** | Sum of topological distance between the vertices O and I | **D750** | Number of group H attached to C0(sp3) with 3X attached to next C |
| **D381** | Sum of topological distance between the vertices S and S | **D751** | Number of group H attached to C0(sp3) with 4X attached to next C |
| **D383** | Sum of topological distance between the vertices S and F | **D752** | Number of group alcohol |
| **D384** | Sum of topological distance between the vertices S and Cl | **D753** | Number of group phenol or enol or carboxyl OH |
| **D385** | Sum of topological distance between the vertices S and Br | **D754** | Number of group O= |
| **D386** | Sum of topological distance between the vertices S and I | **D755** | Number of group Al-O-Al |
| **D387** | Sum of topological distance between the vertices P and P | **D756** | Number of group Al-O-Ar or Ar-O-Ar or R-O-C=X |
| **D392** | Sum of topological distance between the vertices F and F | **D757** | Number of group Al-NH2 |
| **D393** | Sum of topological distance between the vertices F and Cl | **D758** | Number of group Al2-NH |
| **D394** | Sum of topological distance between the vertices F and Br | **D759** | Number of group Al3-N |
| **D395** | Sum of topological distance between the vertices F and I | **D760** | Number of group Ar-NH2 or X-NH2 |
| **D396** | Sum of topological distance between the vertices Cl and Cl | **D761** | Number of group Ar-NH-Al |
| **D397** | Sum of topological distance between the vertices Cl and Br | **D762** | Number of group Ar-NAl2 |
| **D398** | Sum of topological distance between the vertices Cl and I | **D763** | Number of group RCO-N< or >N-X=X |
| **D399** | Sum of topological distance between the vertices Br and Br | **D764** | Number of group Ar2NH or Ar3N or Ar2N-Al |
| **D402** | Walk count order-01 | **D765** | Number of group R#N or R=ND |
| **D403** | Walk count order-02 | **D768** | Number of group Ar-N=X or X-N=X |
| **D404** | Walk count order-03 | **D770** | Number of group R2S or RS-SR |
| **D405** | Walk count order-04 | **D771** | Number of group R=S |
| **D406** | Walk count order-05 | **D772** | Number of group R-SO-R |
| **D407** | Walk count order-06 | **D773** | Number of group R-SO2-R |
| **D408** | Walk count max-10 steps | **D774** | Unsaturation index weighted by conventional bonds order |
| **D409** | Walk-returning count order-1 | **D775** | Hydrophilic factor index |
| **D410** | Walk-returning count order-2 | **D776** | Aromatic bonds ratio |
| **D411** | Walk-returning count order-3 | **D777** | Molecular regression coefficients surface LogP index |
| **D412** | Walk-returning count order-4 |  |  |

**Section 1. Evaluation of GPCR-A17 MAAP and GPCR-A17 MAAP (Ki-filtered) vs. Per-Receptor Similarity and Random Baseline Performance**

To benchmark the GPCR-A17 MAAP and GPCR-A17 MAAP (Ki-filtered) models against intuitive and interpretable alternatives, we implemented two distinct baselines: a per-receptor frequency-aware random classifier (Baseline a), and a per-receptor similarity-based classifier (Baseline b). These baselines serve as lower-bound expectations that account for receptor-specific distribution and structure–function relationships.

A unified reference dataset was constructed by merging all compounds from the training, validation, and testing splits, yielding a pool of ligands annotated as agonists, antagonists, or modulators for 21 unique GPCR targets. The evaluation set, referred to as the ligand validation set, consisted of novel compounds mapped to 20 receptors (excluding TA_3_R, which lacked eligible ligands). The same ligand validation set was used to evaluate both the GPCR-A17 MAAP ML models and the baseline approaches. For the Ki-filtered models, the reference dataset was restricted to ligands with reported Ki values spanning 19 GPCR targets. The corresponding ligand validation set included novel compounds associated with 18 of these 19 receptors and was consistently used across all model and baseline evaluations.

1. **Baseline a: Per-Receptor Frequency-Aware Random Classifier**

First, we implemented a random baseline that accounts for class imbalance on a per-receptor basis. For each compound in the ligand validation set, we sampled a functional label (agonist, antagonist, or modulator) according to the empirical class distribution of the compound’s receptor in the reference dataset. For example, if a receptor’s known ligands are distributed as 60% antagonists, 30% agonists, and 10% modulators, a random assignment is performed using these probabilities. This baseline serves as a minimal expectation, conditioned on receptor-specific frequencies, allowing us to assess how well any classifier outperforms a stochastic yet receptor-aware assignment. The performance metrics for this random baseline are reported for a direct comparison with GPCR-A17 MAAP and GPCR-A17 MAAP (Ki-filtered) **(Tables S10 and S11).**

***Table S10.*** *Comparison of the classification performance between the full XGBoost-based metamodel (GPCR-A17 MAAP) and the per-receptor random baseline for each functional class on the independent ligand validation set. The reported metrics include recall, precision, and F1-score, which are shown as the mean performance of the GPCR-A17 MAAP model. The best value for each metric per class is highlighted in bold type. GPCR: G protein-coupled receptor; MAAP: modulator, agonist, and antagonist predictor.*

|  | **Method** | **Recall** | **Precision** | **F1 Score** |
| --- | --- | --- | --- | --- |
| Antagonist | Random Baseline | 0.5429 | 0.5444 | 0.5436 |
|  | GPCR-A17 MAAP | **0.9000** | **0.7202** | **0.8001** |
| Agonist | Random Baseline | 0.3744 | 0.3668 | 0.3706 |
|  | GPCR-A17 MAAP | **0.7190** | **0.7317** | **0.7253** |
| Modulator | Random Baseline | 0.1837 | 0.1875 | 0.1856 |
|  | GPCR-A17 MAAP | **0.3565** | **0.8319** | **0.4991** |

***Table S11.*** *Comparison of classification performance between the Ki-filtered LightGBM-based metamodel (GPCR-A17 MAAP (Ki-filtered)) and the per-receptor random baseline for each functional class in the independent ligand validation set. The reported metrics include recall, precision, and F1-score, and are shown as the mean performance of the GPCR-A17 MAAP (Ki-filtered) model. The best value for each metric per class is highlighted in bold type. GPCR: G protein-coupled receptor; MAAP: modulator, agonist, and antagonist predictor; Ki: Inhibition Constant.*

|  | **Method** | **Recall** | **Precision** | **F1 Score** |
| --- | --- | --- | --- | --- |
| Antagonist | Random Baseline | 0.5820 | 0.6183 | 0.5996 |
|  | GPCR-A17 MAAP (Ki-Filtered) | **0.8805** | **0.8669** | **0.8736** |
| Agonist | Random Baseline | 0.2990 | 0.3537 | 0.3240 |
|  | GPCR-A17 MAAP (Ki-Filtered) | **0.7526** | **0.7511** | **0.7518** |
| Modulator | Random Baseline | 0.3378 | 0.2404 | 0.2809 |
|  | GPCR-A17 MAAP (Ki-Filtered) | **0.7405** | **0.7851** | **0.7622** |

1. **Baseline b: Per-Receptor Similarity-Based Classifier**

To evaluate the structural similarity as a predictive proxy, we implemented a non-parametric similarity-based classifier using ECFP4 fingerprints (Morgan radius = 2 and 1024 bits), which captures substructure-level features commonly used in cheminformatics. For each compound in the ligand validation set, we identified the corresponding receptor and retrieved all the labelled ligands for that receptor from the reference dataset. We then computed the pairwise Tanimoto similarity between the compound and each receptor-specific reference ligand. Next, we calculated the average similarity of each functional class (agonist, antagonist, modulator) to ligands and assigned the compound to the class with the highest average similarity. The performance metrics for this similarity baseline are reported per class for a direct comparison with GPCR-A17 MAAP and GPCR-A17 MAAP (Ki-filtered) **(Tables S12 and S13).**

***Table S12.*** *Comparison of the classification performance between the full XGBoost-based metamodel (GPCR-A17 MAAP) and the per-receptor similarity baseline for each functional class in the independent ligand validation set. The reported metrics include recall, precision, and F1-score, which are shown as the mean performance of the GPCR-A17 MAAP model. The best value for each metric per class is highlighted in bold type. GPCR: G protein-coupled receptor; MAAP: modulator, agonist, and antagonist predictor.*

|  | **Method** | **Recall** | **Precision** | **F1 Score** |
| --- | --- | --- | --- | --- |
| Antagonist | Similarity Baseline | 0.6171 | 0.7152 | 0.6626 |
|  | GPCR-A17 MAAP | **0.9000** | **0.7202** | **0.8001** |
| Agonist | Similarity Baseline | 0.7179 | 0.6167 | 0.6635 |
|  | GPCR-A17 MAAP | **0.7190** | **0.7317** | **0.7253** |
| Modulator | Similarity Baseline | **0.4354** | 0.3926 | 0.4129 |
|  | GPCR-A17 MAAP | 0.3565 | **0.8319** | **0.4991** |

***Table S13.*** *Comparison of classification performance between the Ki-filtered LightGBM-based metamodel (GPCR-A17 MAAP (Ki-filtered)) and the per-receptor similarity baseline for each functional class in the independent ligand validation set. The reported metrics include recall, precision, and F1-score, and are shown as the mean performance of the GPCR-A17 MAAP (Ki-filtered) model. The best value for each metric per class is highlighted in bold type. GPCR: G protein-coupled receptor; MAAP: modulator, agonist, and antagonist predictor; Ki: Inhibition Constant.*

|  | **Method** | **Recall** | **Precision** | **F1 Score** |
| --- | --- | --- | --- | --- |
| Antagonist | Similarity Baseline | 0.6562 | 0.8195 | 0.7289 |
|  | GPCR-A17 MAAP (Ki-Filtered) | **0.8805** | **0.8669** | **0.8736** |
| Agonist | Similarity Baseline | 0.6907 | 0.6204 | 0.6537 |
|  | GPCR-A17 MAAP (Ki-Filtered) | **0.7526** | **0.7511** | **0.7518** |
| Modulator | Similarity Baseline | **0.7432** | 0.4825 | 0.5851 |
|  | GPCR-A17 MAAP (Ki-Filtered) | 0.7405 | **0.7851** | **0.7622** |


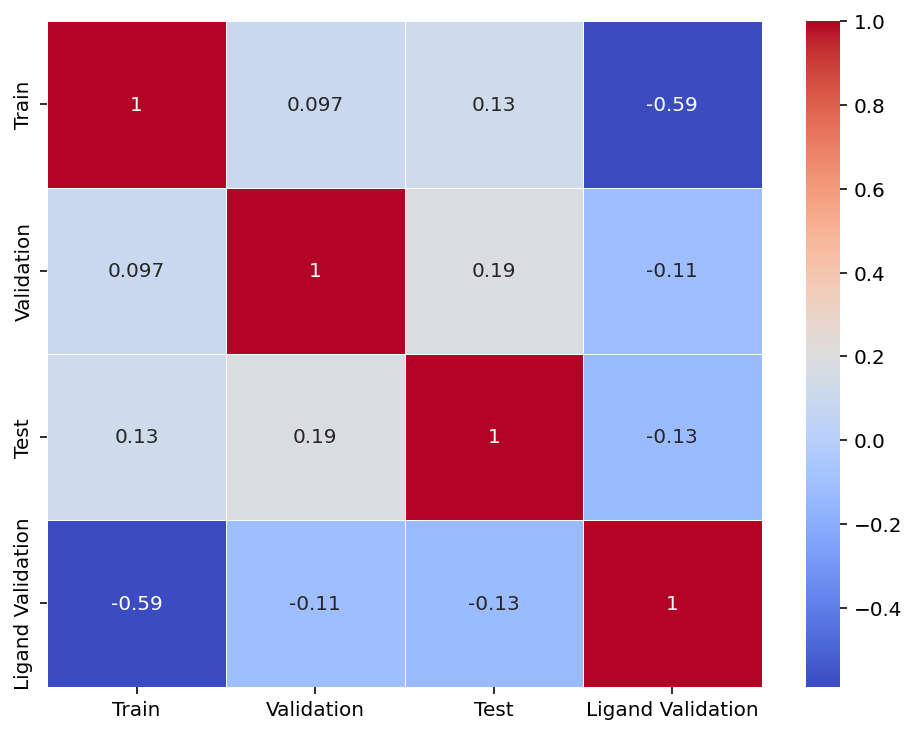


***Figure S1.*** ***Scaffold overlap correlation heatmap between training, validation, testing, and independent ligand validation sets for the full dataset.***

***Table S14.* *Scaffold distribution across different dataset splits, including the number of total scaffolds, unique scaffolds, scaffold frequency distribution, Shannon entropy (H), and normalised Shannon entropy (H_normalized_****)****.*** *Total Scaffolds: The total number of scaffolds present in each dataset; Unique Scaffolds: The number of distinct scaffolds in the respective dataset; Max Scaffold Frequency: The highest occurrence of any single scaffold within the dataset; Min Scaffold Frequency: The lowest occurrence of any scaffold; Shannon Entropy: A measure of scaffold diversity, where higher values indicate a more even scaffold distribution. Normalised Shannon Entropy (H_normalized_): A value of 1 represents the highest uncertainty (maximum scaffold diversity), indicating an even distribution of scaffolds. A value of 0 represents no uncertainty, indicating that the dataset is highly imbalanced or dominated by a single scaffold.*

| *Dataset* | *Total Scaffolds* | *Unique Scaffolds* | *Unique/Total Ratio* | *Max Scaffold Frequency* | *Min Scaffold Frequency* | *Shannon Entropy (H)* | *Normalized Shannon Entropy (H_normalized_)* |
| --- | --- | --- | --- | --- | --- | --- | --- |
| *Training* | *4,981* | *1,297* | *0.2604* | *139* | *1* | *9.3316* | *0.9024* |
| *Validation* | *623* | *371* | *0.5955* | *17* | *1* | *8.1086* | *0.9500* |
| *Testing* | *623* | *381* | *0.6116* | *24* | *1* | *8.1158* | *0.9466* |
| *Independent Ligand Validation* | *692* | *485* | *0.7009* | *40* | *1* | *8.4560* | *0.9478* |


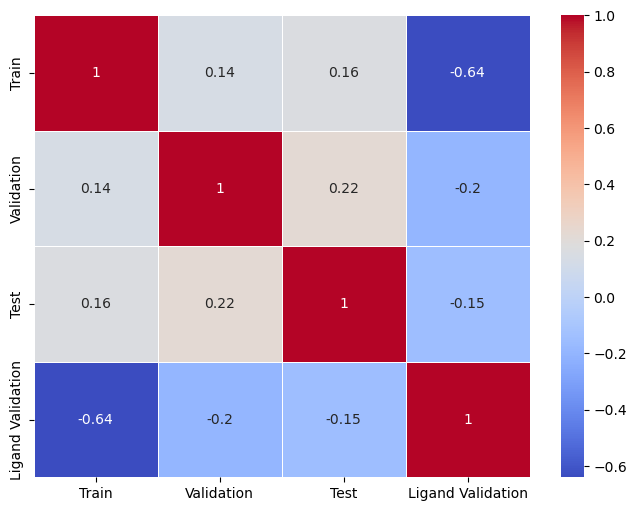


***Figure S2. Scaffold overlap correlation heatmap between training, validation, testing, and independent ligand validation sets for the Ki-filtered dataset.***

***Table S15.* *Scaffold distribution across different dataset splits, including the total number of scaffolds, unique scaffolds, scaffold frequency distribution, Shannon entropy (H), and normalised Shannon entropy (H_normalized_****)* ***(Ki-filtered dataset).*** *Total Scaffolds: The total number of scaffolds present in each dataset; Unique Scaffolds: The number of distinct scaffolds in the respective dataset; Max Scaffold Frequency: The highest occurrence of any single scaffold within the dataset; Min Scaffold Frequency: The lowest occurrence of any scaffold; Shannon Entropy: A measure of scaffold diversity, where higher values indicate a more even scaffold distribution. Normalised Shannon Entropy (H_normalized_): A value of 1 represents the highest uncertainty (maximum scaffold diversity), indicating an even distribution of scaffolds. A value of 0 represents no uncertainty, indicating that the dataset is highly imbalanced or dominated by a single scaffold.*

| *Dataset* | *Total Scaffolds* | *Unique Scaffolds* | *Unique/Total Ratio* | *Max Scaffold Frequency* | *Min Scaffold Frequency* | *Shannon Entropy (H)* | *Normalized Shannon Entropy (H_normalized_)* |
| --- | --- | --- | --- | --- | --- | --- | --- |
| *Training* | *3,077* | *783* | *0.2545* | *72* | *1* | *8.7068* | *0.9057* |
| *Validation* | *385* | *246* | *0.6390* | *12* | *1* | *7.5846* | *0.9549* |
| *Testing* | *385* | *249* | *0.6468* | *13* | *1* | *7.6107* | *0.9561* |
| *Independent Ligand Validation* | *427* | *286* | *0.6698* | *13* | *1* | *7.8082* | *0.9569* |
